# Nonequivalent lethal equivalents: Models and inbreeding metrics for unbiased estimation of inbreeding load

**DOI:** 10.1111/eva.12713

**Published:** 2018-10-23

**Authors:** Pirmin Nietlisbach, Stefanie Muff, Jane M. Reid, Michael C. Whitlock, Lukas F. Keller

**Affiliations:** ^1^ Department of Evolutionary Biology and Environmental Studies University of Zurich Zurich Switzerland; ^2^ Department of Zoology University of British Columbia Vancouver BC Canada; ^3^ School of Biological Sciences University of Aberdeen Aberdeen UK; ^4^ Zoological Museum University of Zurich Zurich Switzerland

**Keywords:** conservation biology, generalized linear (mixed) models, genomics, inbreeding coefficients, inbreeding depression, pedigree, runs of homozygosity

## Abstract

Inbreeding depression, the deterioration in mean trait value in progeny of related parents, is a fundamental quantity in genetics, evolutionary biology, animal and plant breeding, and conservation biology. The magnitude of inbreeding depression can be quantified by the inbreeding load, typically measured in numbers of lethal equivalents, a population genetic quantity that allows for comparisons between environments, populations or species. However, there is as yet no quantitative assessment of which combinations of statistical models and metrics of inbreeding can yield such estimates. Here, we review statistical models that have been used to estimate inbreeding load and use population genetic simulations to investigate how unbiased estimates can be obtained using genomic and pedigree‐based metrics of inbreeding. We use simulated binary viability data (i.e., dead versus alive) as our example, but the concepts apply to any trait that exhibits inbreeding depression. We show that the increasingly popular generalized linear models with logit link do not provide comparable and unbiased population genetic measures of inbreeding load, independent of the metric of inbreeding used. Runs of homozygosity result in unbiased estimates of inbreeding load, whereas inbreeding measured from pedigrees results in slight overestimates. Due to widespread use of models that do not yield unbiased measures of the inbreeding load, some estimates in the literature cannot be compared meaningfully. We surveyed the literature for reliable estimates of the mean inbreeding load from wild vertebrate populations and found an average of 3.5 haploid lethal equivalents for survival to sexual maturity. To obtain comparable estimates, we encourage researchers to use generalized linear models with logarithmic links or maximum‐likelihood estimation of the exponential equation, and inbreeding coefficients calculated from runs of homozygosity, provided an assembled reference genome of sufficient quality and enough genetic marker data are available.

## 1 INTRODUCTION

Inbreeding depression, the deterioration in mean trait value in progeny of related parents (Crow & Kimura, 1970, chapter 3), is a fundamental quantity in genetics, evolutionary biology, animal and plant breeding, and conservation biology (Charlesworth & Willis, 2009; Hedrick & Kalinowski, 2000; Kristensen & Sorensen, 2005; Wright, 1977). Conceptual and practical advances in these disciplines require accurate and robust estimates of the magnitude of inbreeding depression that can be compared among different traits, among sets of individuals of different ages and sexes, and among different environments, populations or species (Armbruster & Reed, 2005; Fox & Reed, 2010; Hoeck, Wolak, Switzer, Kuehler, & Lieberman, 2015; Kruuk, Sheldon, & Merilä, 2002; Leroy, 2014; Waller, Dole, & Bersch, 2008). These goals in turn require widespread adoption of a standard estimator of the magnitude of inbreeding depression that is unbiased, quantitatively comparable and firmly rooted in population genetic theory.

One such estimator is the inbreeding load, *B*, measured as the negative slope of a regression of the logarithm of a trait on inbreeding coefficient *F* (Charlesworth & Charlesworth, 1987; Charlesworth & Willis, 2009; Keller & Waller, 2002). Inbreeding load in viability (i.e., survival versus mortality) is measured in units of “lethal equivalents,” where one lethal equivalent corresponds to a group of deleterious alleles that would cause one death on average if made homozygous (Morton, Crow, & Muller, 1956). The number of lethal equivalents can equally be interpreted as the number of deaths that would be expected in a group of hypothetical individuals where each individual carried one deleterious allele in homozygous state (i.e., the group contains as many individuals as there are deleterious alleles; Morton et al., 1956). Hence, one lethal equivalent can correspond to a lethal allele at one locus or to several mildly deleterious alleles at several loci. The concept of lethal equivalents was invented to quantify inbreeding depression in viability (Morton et al., 1956), hence the terminology “lethal.” Throughout our study, we use viability data as example. However, the general approach to quantifying inbreeding load as a logarithmic relationship with *F* can be applied to other fitness components (Charlesworth & Charlesworth, 1987), or indeed to any other trait as long as alleles that improve trait value are, on average, dominant over alleles that reduce trait value or show overdominance (Wolak & Keller, 2014).

In population genetic theory, inbreeding load is defined as

(1) $$B=\sum_{i=1}^{L}q_{i}s_{i}-\sum_{i=1}^{L}q_{i}^{2}s_{i}-2\sum_{i=1}^{L}\left(q_{i}\left[1-q_{i}\right]s_{i}h_{i}\right),$$

where $q_{i}$ is the frequency of the deleterious allele i, $s_{i}$ is its deleterious effect when homozygous, $h_{i}$ is the dominance coefficient, and the sum is taken over all L biallelic loci at which deleterious alleles can occur (Morton et al., 1956).

Morton et al.'s (1956) fundamental insight was that inbreeding load *B* for trait *y* can be estimated in the absence of information on $q_{i}$, $s_{i}$ and $h_{i}$ simply as the slope of a weighted regression of $-\log_{e}\left(y\right)$ on F, that is

(2) $$-\log_{e}\left(y\right)=A+BF,$$

with individuals pooled into groups of similar *F*, and where *A* is the intercept and *y* the expected value of the trait for that level of *F*. This model is itself rooted in population genetics theory and assumes that effects of different environmental and genetic factors act independently and thus have multiplicative effects that translate into additive effects only on the logarithmic scale (Charlesworth & Charlesworth, 1987). It is therefore important that a logarithmic scale is used.

When data are only available for mean trait values of known outbred ($y_{0}$) and inbred individuals ($y_{F}$) with a single known level of F, for example offspring of selfing or full‐sibling mating generated in a breeding design, the inbreeding load can be estimated as

(3) $$B=-\log_{e}\left(y_{F}/y_{0}\right)/F$$

(Charlesworth & Charlesworth, 1987; Lynch & Walsh, 1998, p. 278). Such breeding designs are hard to impose in wild, free‐living populations or captive populations of endangered animals, but comparable and unbiased estimates of inbreeding load from such populations are key to understanding evolutionary dynamics (Kokko & Ots, 2006) and deciding population management strategies (Caballero, Bravo, & Wang, 2017a,b; Theodorou & Couvet, 2017). Morton et al.'s (1956) regression model (equation 2) provides a conceptually elegant and theoretically well‐founded approach for estimating inbreeding load that can be applied given a range of naturally occurring *F* values. However, implementation has not been without difficulties that have impeded widespread adoption despite recognition of its useful properties (Keller & Waller, 2002). Indeed, relatively few wild population studies have so far explicitly reported estimates of inbreeding load (Table 1).

**Table 1:** Estimates of inbreeding load from wild vertebrate populations obtained with unbiased statistical models. All studies calculated inbreeding coefficients from pedigree data (i.e., *F*_ped_). The model used to estimate inbreeding load is coded 1 for logarithmic regression or class comparisons similar to the model proposed by Morton et al. (1956) or 2 for maximum‐likelihood estimation of an exponential relationship. The life stage column indicates the time frame over which survival was assessed. The next five columns list haploid inbreeding load *B* for traits assigned to the following life stages: survival in juveniles (Juv.), survival until approximately half the age of sexual maturity (50%), survival until approximately sexual maturity (100%), survival in adults (Ad.) and reproductive traits (Rep.). The last column lists the publication that reported the inbreeding load or that reported the data used to calculate the inbreeding load

Species	Model	Life stage (survival or reproduction)	Juv.	50%	100%	Ad.	Rep.	Publication
Cactus finch	2	8 days to 1 year			4.3			Keller, Grant, Grant, and Petren (2002)
Chatham Island black robin	1	Fledging to 1 year*	1.4					Kennedy et al. (2014)
Collared flycatcher	2	Survival to 1 year			7.5			Kruuk et al. (2002)
Great tit	1	Egg to hatching*	1.0					van Noordwijk and Scharloo (1981)
Great tit	1	Egg to fledging*	0.9					van Noordwijk and Scharloo (1981)
Great tit	1	Egg to hatching	0.4					Szulkin, Garant, McCleery, and Sheldon, (2007)
Great tit	1	Hatching to fledging	0.4					Szulkin et al. (2007)
Great tit	1	Fledging to recruitment	1.3					Szulkin et al. (2007)
Great tit	1	Egg to recruitment			2.1			Szulkin et al. (2007)
Large ground finch	2	8 days to 1 year			4.5			Grant, Grant, and Petren (2001), Keller et al. (2002)
Medium ground finch	2	8 days to 1 year			0.0			Keller et al. (2002)
Mexican jay	1	Nestling to 1 year*		5.6				Brown and Brown (1998)
Moorhen	1	Egg to hatching*	2.2					McRae (1996)
North Island robin	2	Fledging to 1 year			4.1			Jamieson, Tracy, Fletcher, and Armstrong (2007)
Song sparrow	1	Egg to 24 days	1.4					Keller (1998)
Song sparrow	1	24 days to 1 year	1.3					Keller (1998)
Song sparrow	1	Egg to 1 year			2.7			Keller (1998)
Song sparrow	1	Fitness (survival and reproduction)					24.6	Wolak, Arcese, Keller, Nietlisbach, and Reid (2018)
Golden lion tamarin	1	To 24 months*			2.8			Dietz, Baker, and Ballou (2000)
Red deer	2	To 1 year		4.4				Walling et al. (2011)
White‐footed mouse	1	ca. 117–138 days				6.3		Jimenez, Hughes, Alaks, Graham, and Lacy (1994)
White‐footed mouse	1	Weekly adult survival				2.3		Jimenez et al. (1994)
Wolf	1	Conception to first winter*	3.0					Liberg et al. (2005)

The estimates for traits marked with an asterisk * are based on our reanalysis of available data. Rationales and methods are described in the R code in the Supporting Information, which also explains why some estimates are omitted. The high estimate of Kruuk et al. (2002) is based on a large data set, but that only includes 22 inbred pairings. Jimenez et al. (1994) estimated adult survival across a 3‐week period (approximately 117–138 days of age), which appears to be the period leading to the largest difference between inbred and outbred individuals (their Figure 2).

One primary problem is that $-\log_{e}\left(y\right)$ is undefined for any level of inbreeding with a trait mean of zero (e.g., zero survivors), meaning that model 2 cannot be directly fitted across all data. Multiple alternative statistical models have consequently been advocated (Table 2). Templeton and Read (1983, 1984) suggested a small sample size correction given group means of zero, but this introduces its own bias (Kalinowski & Hedrick, 1998; Lacy, 1997; Willis & Wiese, 1997). Kalinowski and Hedrick (1998) proposed a model that avoids the issue of undefined logarithms by directly fitting the exponential model $y_{F}=y_{0}e^{-\mathrm{BF}}$. Kruuk et al. (2002) extended this model to allow for heterogeneity in outbred survival and inbreeding load among years. García‐Dorado, Wang, and López‐Cortegano (2016) also developed software to fit this model to individual‐level data. Glémin, Vimond, Ronfort, Bataillon, and Mignot (2006) used generalized linear models (GLMs) with a logarithmic link to estimate the regression slope *B*, pooling groups of individuals with similar levels of inbreeding. As an alternative that does not require calculation of group means, Armstrong and Cassey (2007) and Grueber, Nakagawa, Laws, and Jamieson (2011) suggested the use of GLMs and generalized linear mixed models (GLMMs) with various link functions and error distributions. As an alternative to the conditional GLMMs, Fredrickson, Siminski, Woolf, and Hedrick (2007) used generalized estimating equations (GEE) to obtain marginal estimates of the number of lethal equivalents. These GLMM and GEE models can easily be applied to individual survival data and, in principle, readily allow estimation of variation in inbreeding depression across ages, sexes or environments. Additional but more rarely used models can be found in Makov and Bittles (1986), Ralls, Ballou, and Templeton (1988), Lee, Lascoux, and Nordheim (1996), Lascoux and Lee (1998) or Hedrick, Hellsten, and Grattapaglia (2016). However, as we will show, some of these models do not preserve the population genetic assumptions (additivity on a logarithmic scale) underlying Morton et al.'s (1956) original derivation and, hence, do not yield comparable unbiased estimates of the inbreeding load.

**Table 2:** Summary of models for estimation of inbreeding load. The names of these models are used in Figure 1. Details for all models are described in Supporting Information 1, and the models are illustrated in Figure S4 in Supporting Information 1. For the model “GLM logit‐link,” we used *F *= 0 and *F *= 0.25 for predictions, but see Supporting Information 1 for a discussion of the effects of the arbitrary choice of these levels

Name	Data structure	Estimation of inbreeding load	References
Morton et al.	Survival rate for classes of *F*	Slope of a weighted regression of mean survival rate on *F*	Morton et al. (1956)
Morton & TR	Survival rate for classes of *F*	Same as Morton et al., but with a correction for small sample size	Templeton and Read (1983, 1984)
Exponent. ML	Individual survival (this study) or classes of *F*	Estimation of *y*_F_ = *y*_0_ *e* ^−BF^ with *y*_0_ = *e* ^−A^ by maximizing the likelihood	Kalinowski and Hedrick (1998)
GLM logit‐link	Individual survival	Fit a generalized linear (mixed) model with binomial errors and logit link function, then use predictions from this model for two levels of *F* (typically *F *= 0 and *F *= 0.25) in equation 3 to obtain inbreeding load	Grueber et al. (2011)
GLM log‐link	Individual survival	Slope (on latent scale) of a generalized linear (mixed) model with Poisson errors and logarithmic link	after Zou, 2004

All these models (Table 2) have in common that they require some metric of the inbreeding coefficient, *F*, of focal individuals (Table 3). Pedigrees allow estimation of inbreeding coefficients (*F*_ped_) that measure the *expected* amount of identity by descent of an individual (Wright, 1969, chapter 7). However, Mendelian sampling and linkage cause *realized* identity by descent to deviate from its expectation (Franklin, 1977; Hill & Weir, 2011; Knief, Kempenaers, & Forstmeier, 2017; Leutenegger et al., 2003; Stam, 1980). Further, wild population pedigrees usually encompass limited numbers of generations and typically contain errors and missing data which can cause bias and error in *F*_ped_ (Knief et al., 2015; Wang, 2014). Recent developments in DNA sequencing technologies and resulting genomic data are now opening opportunities to quantify *realized* identity by descent and hence quantify inbreeding load through genomic rather than traditional pedigree‐based approaches (Curik, Ferenčaković, & Sölkner, 2014; Hoffman et al., 2014; Kardos, Taylor, Ellegren, Luikart, & Allendorf, 2016; Keller, Visscher, & Goddard, 2011). Several methods to estimate inbreeding coefficients from genomic data are available. In the absence of an assembled reference genome, *F* can be quantified as a deviation in observed heterozygosity from its expectation based on Hardy–Weinberg equilibrium (Wang, 2014, 2016). If an assembled reference genome is available, chromosomal regions can be identified that are homozygous in an individual, and the proportion of the genome in such “runs of homozygosity” is then used to calculate *F*_ROH_ (McQuillan et al., 2008). Because *F*_ROH_ is calculated as a proportion, it ranges from 0 to 1, as does *F*_ped_, while metrics based on deviation from Hardy–Weinberg equilibrium include positive and negative values (Table 3). Thus, the various estimators of inbreeding differ not only in data requirements and meaning, but also in some of their properties, such as range, mean and variance. These differences may affect resulting estimates of inbreeding load (Kardos, Nietlisbach, & Hedrick, 2018; Yengo et al., 2017).

**Table 3:** Properties of the different metrics of *F*. The theoretically possible range and the expected mean are listed. Observed mean, variance, minimum and maximum were calculated for each of the 280 demes simulated in this study, and their means (e.g., the mean of all 280 observed minima) are reported in this table

Metric of F	Based on	Possible range	Expected mean	Observed mean	Observed variance	Observed minimum	Observed maximum
*F*_ped_	Pedigree	[0, 1]	Positive	0.0878	0.0016	0.0005	0.3276
*F*_ROH_	Runs of homozygosity	[0, 1]	Positive	0.0925	0.0024	0.0002	0.4095
*F*_H_	Hardy–Weinberg expectation	(−∞, ∞)	0	−0.0005	0.0039	−0.1625	0.3560
*F*_alt_	Hardy–Weinberg expectation and weight on rare homozygotes	(−∞, ∞)	0	−0.0047	0.0022	−0.0806	0.3432

Despite the need for comparable and unbiased estimates of inbreeding load across diverse natural populations and the increasing diversity of available statistical models (Table 2) and metrics of *F* (Table 3), there is as yet no quantitative assessment of which combinations of models and metrics can yield the requisite estimates. Such assessments must themselves be consistent with underlying population genetic theory. Accordingly, we used population genetic simulations to investigate how unbiased measures of inbreeding load can be obtained using genomic and pedigree‐based estimates of inbreeding and thereby provide a generally applicable roadmap for future studies.

## 2 MATERIALS AND METHODS

We conducted two sets of independent simulations in this study. First, we used phenotypic simulations where survival (i.e., a binary variable representing dead or alive individuals) was a direct function of *F* to explore the different statistical models used to estimate inbreeding load. Second, we used genetically explicit simulations of a metapopulation to investigate the performance of different pedigree‐based and genomic metrics of *F*. For these genetic simulations, survival was determined by loci with deleterious mutations. For the first set of phenotypic simulations, we used values of *F* from individuals of one of the demes of the metapopulation. For this reason, we first describe the general set‐up of the metapopulation simulations, then the set of phenotypic simulations (where *F* directly affects survival) and finally the set of genetically explicit simulations (where survival is affected by simulated genotypes).

### 2.1 Genetic simulations of metapopulations

We conducted genetically explicit simulations using Nemo v2.3.46r4 (Guillaume & Rougemont, 2006). To represent patterns of inbreeding that can emerge in natural vertebrate populations, simulations were loosely inspired by a song sparrow (*Melospiza melodia*) metapopulation on the Gulf Islands in British Columbia, Canada, which is known to express considerable among‐individual variation in the degree of inbreeding and to show inbreeding depression in fitness traits (Keller, 1998; Nietlisbach et al., 2017; Reid et al., 2014; Smith, Keller, Marr, & Arcese, 2006; Wilson & Arcese, 2008; Table 1).

We simulated 30 demes of up to 200 diploid individuals each for 5,000 non‐overlapping generations. Demes were connected through dispersal in an island model with a mean of 1.2 surviving immigrants per deme and generation. Thus, while some immigrants could be related to individuals in the receiving deme (if their anecestors had previously emigrated), they are unlikely to be closely related.

Individuals within a deme paired randomly, and each female produced a number of offspring sampled from a Poisson distribution with mean 10. Offspring paternity was assigned with an extra‐pair paternity rate of 28% (as in song sparrows; Sardell, Keller, Arcese, Bucher, & Reid, 2010) sired by random males in the same deme, thereby generating a pedigree structure typical of many natural populations with numerous maternal and paternal half‐sibs as well as full‐sibs (e.g., Germain, Arcese, & Reid, 2018).

After reproduction, each deme was culled to 200 individuals through random mortality, followed by random dispersal without spatial structure. Genotypes (see below) of all individuals in the metapopulation were recorded. Viability selection was then applied using the survival probability of each of the 200 individuals as determined by their genotypes at loci with deleterious alleles (see below). Viability selection thus reduced the number of adult individuals to below 200 per deme, but this order of life cycle events ensured that viability selection was the only nonrandom source of mortality.

Analyses were performed for each of 28 demes separately (simulation output from two demes was accidentally deleted) from each of 10 replicate simulation runs, yielding a total of 280 estimates. Immigrants were excluded from analyses as is often done in field studies where *F* of immigrants is typically unknown due to missing pedigree information or unknown allele frequencies in their deme of origin.

The simulated diploid genome mimicked a great tit (*Parus major*) genome with recombination map length per chromosome taken as the mean of both populations measured by van Oers et al. (2014). We distributed 49,998 biallelic neutral loci and 2,500 biallelic loci with deleterious alleles (termed “deleterious loci”) onto chromosomes proportional to the physical size in base pairs of the 28 autosomes with known attributes (Laine et al., 2016). Nemo then distributed these loci randomly within the chromosomes (see also Nietlisbach et al., 2017).

Neutral loci were initialized by randomly and independently allocating one of two alleles at each homologous position. This resulted in binomially distributed allele frequencies around an expected frequency of 0.5 at the simulation start (49,828 loci or 99.66% were on average polymorphic among the analysed individuals at the end of the simulation). Loci were biallelic to match the most frequently observed pattern for intraspecific single nucleotide polymorphisms.

Compared to the neutral loci, a smaller fraction (2,122 loci or 84.88%) of deleterious loci were on average polymorphic among the analysed individuals, as expected with selection against deleterious alleles and inbreeding exposing recessive deleterious alleles (i.e., purging). Deleterious loci acted independently and therefore contributed multiplicatively to individual survival probability by factors of 1, 1 − *h*_i_ *s*_i_ and 1 − *s*_i_ per locus that was homozygous for the beneficial allele, heterozygous and homozygous for the deleterious allele, respectively. Individual survival probabilities determined how likely an individual was to survive to adulthood. We recorded whether individuals survived or died in the simulations, and this binary measure was used to compare the performance of different metrics of *F* for estimation of inbreeding load (see below).

Our simulations follow the genetic model of Morton et al. (1956) by assuming no epistasis. We also did not simulate overdominant loci. We will revisit these assumptions in the Discussion. Selection coefficients $s_{i}$ were drawn from an exponential distribution with mean $\overset{¯}{s}\;=\;0.03,$ a value in the middle of empirical estimates (reviewed by Wang, Hill, Charlesworth, & Charlesworth, 1999). Dominance coefficients $h_{i}$ were determined by Nemo with a function that assigned smaller dominance coefficients to alleles with larger deleterious effects: $h_{i}\;=\;0.5\exp\left(\log\left(2\overset{¯}{h}\right)s_{i}/\overset{¯}{s}\right)$ with $\overset{¯}{h}\;=\;0.1$ (Wang et al., 1999). Due to the exponential distribution of $s_{i}$, the simulated mean dominance coefficient was 0.18, a value close to empirical mean estimates of 0.2–0.4 (reviewed by Wang et al., 1999) or 0.1–0.3 (reviewed by Lynch & Walsh, 1998, p. 286). The resulting distributions of $s_{i}$ (range from 1.25 × 10^−5^ to 0.22) and $h_{i}$ (range from 2.94 × 10^−6^ to 0.50), and their relationship are shown in Figure S1 in Supporting Information 1.

Mutation rate at neutral and deleterious loci was set to 0.0002. Mutation rate and number of deleterious loci were chosen in conjunction so that a diploid individual would experience on average one new deleterious mutation, a value compatible with empirical data (Lynch & Walsh, 1998, p. 351; Wang et al., 1999). Due to constraints of Nemo, neutral loci could mutate from either allele to the other, whereas deleterious loci could only mutate to the deleterious allele. Following Wang (2015), all deleterious loci were initialized at the same equilibrium allele frequency expected in a large population, calculated with $\overset{¯}{s},\overset{¯}{h}$, and the mutation rate (Crow & Kimura, 1970, equation 6.2.6).

Simulations were run for 5,000 generations, by which time they had reached near‐equilibrium of genetic drift, migration, mutation and selection as shown by stabilized mean heterozygosity and allele frequency distributions (data not shown). The genotypes of individuals conceived in generations 4,996–4,999 were recorded, yielding a sample size of 788 individuals per deme on average. The cut‐off was the second last of 5,000 simulated generations because survival was not simulated for last‐generation individuals. The simulations resulted in a mean inbreeding load of 1.83, with a range of 1.63 to 2.10, and a standard deviation of 0.08 lethal equivalents across the analysed data sets.

Pedigree‐based inbreeding coefficients *F*_ped_ (Wright, 1969; chapter 7) were calculated based on the previous 20 generations of the metapopulation pedigree (i.e., since generation 4,976, yielding a pedigree of up to 25 generations) using the R package *pedigreemm* (Vazquez, Bates, Rosa, Gianola, & Weigel, 2010). Three genomic metrics of *F* were calculated using neutral loci (Table 3). Although some loci with deleterious effects may be part of empirical data sets, we excluded them here because realistic genomic data sets are unlikely to contain all deleterious loci and many of them would be excluded due to minor allele frequency cut‐offs.

The first genomic metric, *F*_H_ (called *F*_HOM_ by Yengo et al., 2017), quantifies inbreeding as a deviation in homozygosity from its Hardy–Weinberg expectation given allele frequencies calculated from the set of individuals for which inbreeding was estimated (Keller et al., 2011). Individuals with negative *F*_H_ are more heterozygous than the average individual under Hardy–Weinberg expectations. Consequently, *F*_H_ must be interpreted as a correlation rather than a probability of identity by descent (Wang, 2014). We calculated *F*_H_ in R v3.2.3 (R Core Team 2015), with verification in PLINK v1.90b4.3 (Purcell et al., 2007).

The second metric *F*_alt_ is similar to *F*_H_ in that it also provides a metric of inbreeding relative to reference allele frequencies, but it differs in that homozygous genotypes are weighted with the inverse of their allele frequency (Yang et al., 2010). Thus, rare homozygous genotypes contribute more to *F*_alt_ than common homozygous genotypes (Keller et al., 2011). We calculated *F*_alt_ in R, with verification using the software for genome‐wide complex trait analysis GCTA v1.26.0 (Yang, Lee, Goddard, & Visscher, 2011). We call this metric *F*_alt_ following Keller et al. (2011); it is called ${{\hat{F}}_{i}}^{\mathrm{III}}$ by Yang et al. (2011), $F_{\mathrm{GRM}}$ by Huisman, Kruuk, Ellis, Clutton‐Brock, and Pemberton (2016) and Bérénos, Ellis, Pilkington, and Pemberton (2016), and *F*_UNI_ by Yengo et al. (2017).

The third metric *F*_ROH_ measures inbreeding as the proportion of the genome that is found in runs of homozygosity (McQuillan et al., 2008). Runs of homozygosity decrease in length with the number of generations *g* since a common ancestor, with an exponential distribution around a mean length *L* of 1/(2g) Morgans (Fisher, 1954; Howrigan, Simonson, & Keller, 2011; Keller et al., 2011). The simulated 28 chromosomes had a total recombination map length of 18.81 Morgans, a genome size of 920 Mega base pairs (Mbp) and a mean recombination rate across the whole genome of 2.04 cM/Mbp = 0.0204 M/Mbp. Hence, runs of homozygosity longer than *L *= 1 Mbp are on average due to coalescence occurring <24.5 generations ago because g=1/(2·L·0.0204). Runs of homozygosity were detected in PLINK in a sliding window of 50 loci (moved in steps of 5), after removing loci that were in strong linkage disequilibrium $(r^{2}>0.9$) to improve accuracy of detecting autozygous runs of homozygosity (Howrigan et al., 2011), and allowing up to one heterozygous locus to account for the possibility of mutation. Stretches of up to 2 Mbp with no loci were allowed to account for random variation in marker density.

For all four metrics of *F*, we calculated mean and variance across all individuals per deme (excluding immigrants).

### 2.2 Comparison of statistical models to estimate inbreeding load

To investigate which of the five focal statistical models for estimation of inbreeding load (Table 2) provided unbiased estimates of *B*, we conducted a set of simulations in R that assumed *F* values were known precisely and were directly affecting fitness. This set of simulations was not genetically explicit, to allow a comparison of statistical models without adding the complexity of potential biases in metrics of *F* that could arise if survival probability and *F* were both estimated from genetic data. Consequently, the performance of different metrics of *F* as proxies for genotypes at loci with deleterious alleles will be addressed in the second set of (genetic) simulations below. Random errors in *F* or fitness, however, did not affect results here (Figures S2 and S3 in Supporting Information 1). To obtain realistic distributions of *F* values for this set of simulations, we used the *F*_ROH_ values of a single deme simulated in Nemo (791 individuals in total). Using these *F* values as input, we calculated the expected survival probability $\pi_{F}$ for each class of individuals with inbreeding coefficient *F* as

(4) $$\pi_{F}=e^{-A-BF}.$$

We then used $\pi_{F}\;$ to create 791 individual survival events ($y_{F}=0:$ dead, $y_{F}=1:$ alive) by sampling survival events from a Bernoulli distribution with success probability $\pi_{F}$. Hence, the individual survival events $y_{F}$ were Bernoulli distributed with residual variance $\pi_{F}\cdot \left(1-\pi_{F}\right)$ around the expectation $\pi_{F}$. The intercept *A* was set to 0.25 or 0.75, and the slope *B* (i.e., the inbreeding load) was set to 1, 5, 10 or 20. For each combination of *A* and *B*, we simulated 10,000 data sets (of 791 individuals each) and then quantified *B* using each statistical model (Table 2). We applied the method of Morton et al. (1956) to data grouped into similarly sized classes of similar values of *F* as summarized in the introduction, both with and without the small sample size correction proposed by Templeton and Read (1983, 1984). Individual survival was analysed using the maximum‐likelihood approach described by Kalinowski and Hedrick (1998). We also fitted a GLM with binomial errors and logit link function, and used predictions from this model in equation 3 as recommended by Grueber et al. (2011). In addition, we fitted a GLM with Poisson error distribution and logarithmic link function. Although not a commonly used approach, it is known that a GLM with Poisson distribution and logarithmic link function does provide unbiased point estimates for binary data (e.g., survival versus mortality) and usually avoids convergence problems that may occur with binomial errors and logarithmic link function. However, standard confidence intervals from a Poisson GLM are typically too large, yet this issue can be resolved by using the so‐called sandwich estimator, a robust error variance estimation procedure (Zou, 2004; Supporting Information 1). For each model and combination of *A* and *B,* we extracted the estimated mean *B* and the 2.5% and 97.5% quantiles across the 10,000 data sets. These simulations directly compare the performance of the different statistical models to estimate inbreeding load (Table 2) using a realistic distribution of *F* values. Further details are provided in Supporting Information 1, along with an illustrated example (FigureS4).

### 2.3 Comparison of effects of metrics of *F* on estimates of inbreeding load

The above analyses showed that a Poisson GLM with logarithmic link provides reliable estimates of inbreeding load (see Results). Therefore, to compare the effects of the four different metrics of *F* on estimates of inbreeding load, we used this statistical model to regress individual survival on *F*_ped_, *F*_H_, *F*_alt_ or *F*_ROH_ in separate analyses. Contrary to the previous analysis, we here used observed survival from the genetically explicit Nemo simulations. Thus, in this analysis, survival probability was determined by individual genotypes at loci with deleterious alleles (see above for details), and neutral loci or the pedigree was used to independently measure *F*. We extracted the slope as an estimate of inbreeding load per replicate and the mean and 2.5% and 97.5% quantiles across the 280 replicates (28 demes from 10 simulation runs). We calculated the actual inbreeding load present in the focal deme using equation 1, with allele frequencies $q_{i}$ from the focal generations 4,996–4,999 and selection $s_{i}$ and dominance coefficients $h_{i}$ for each locus as used in the Nemo simulations. This value provided a genetic reference that equals the value that a reliable method should estimate. We considered a metric of *F* to be biased if the difference between actual inbreeding load (calculated using equation 1) and its estimate was different from 0 with a *p*‐value of less than 5%, as assessed using an intercept‐only linear model with that difference for each deme as response variable. We additionally calculated root mean square error (RMSE), which is a combined measure of accuracy and precision.

Although a GLM with Poisson distribution and logarithmic link function provides unbiased point estimates for binary data, a sandwich estimator has to be used to calculate robust standard errors (Zou, 2004; Supporting Information 1 and above). Then, 95% Wald confidence intervals for *B* were estimated as the point estimate ±1.96 times the robust standard error for each deme and metric of *F*. We then quantified the number of replicates in which the confidence interval contained the actual inbreeding load. If a method is unbiased, this proportion should be close to 95%.

Additional analyses to examine the sensitivity of our results and conclusions to pedigree depth (affecting estimation of *F*_ped_), to the set of loci considered (affecting estimation of *F*_H_ and *F*_alt_), to the length of runs of homozygosity (affecting estimation of *F*_ROH_), to the number of individuals considered per deme, and to different filtering of neutral loci with respect to minor allele frequencies and linkage disequilibrium are summarized in Supporting Information 2.

## 3 RESULTS

### 3.1 Comparison of statistical models to estimate inbreeding load

**Figure 1:**
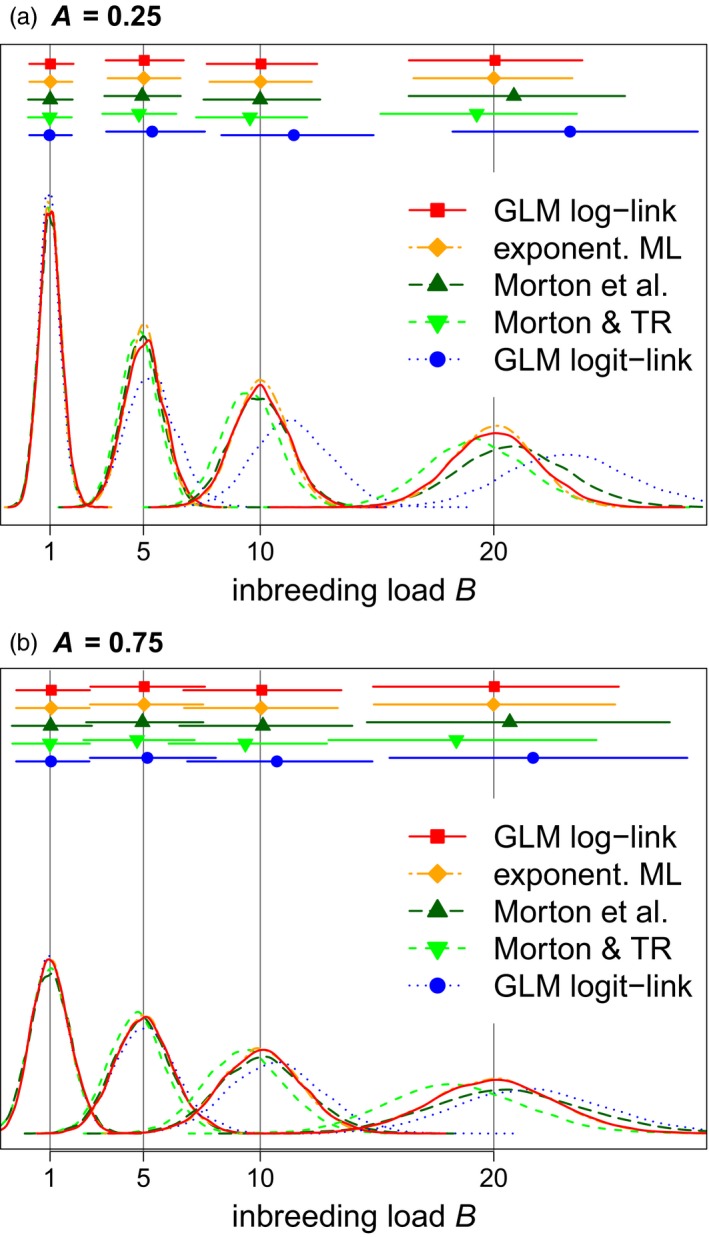
Simulations of 10,000 data sets of survival (binary variable representing dead or alive) for four levels of inbreeding load (B=1,5,10,20), two different intercepts of (a) A = 0.25 or (b) A = 0.75, and 791 individuals with realistic *F* values and binary survival events *y*_F_ sampled with survival probabilities *Π*_F_ = *e* ^−*A*‐*BF*^. We quantified inbreeding load using the models summarized in Table 2 and illustrated in Figure S4 in Supporting Information 1. Inbreeding load was estimated as the slope of a Poisson generalized linear model with logarithmic link function (“GLM log‐link”), with an exponential model (“exponent. ML”), by weighted regression either without (“Morton et al.”) or with the small sample size correction of Templeton and Read (1983, 1984) (“Morton & TR”), and from a binomial generalized linear model with logit link function (“GLM logit‐link”). Probability densities across the 10,000 simulations are shown along the *y*‐axis for each value of *B* in the lower parts of each panel. The estimated means of *B* across 10,000 simulations are indicated by dots along the top of each panel, and the horizontal lines indicate the central 95% range

Fitting the full set of statistical models (Table 2) to the simulated individual survival data showed that only the GLM with logarithmic link function, and the maximum‐likelihood estimation of the exponential equation, provided unbiased estimates of inbreeding load in all cases (Figure 1). These two methods fit essentially identical models in different ways.

Morton et al.'s (1956) regression model substantially underestimated *B* when applying the small sample size correction of Templeton and Read (1983, 1984), confirming previous extensive simulation studies (Kalinowski & Hedrick, 1998; Lacy, 1997; Willis & Wiese, 1997). Without the small sample size correction, Morton et al.'s model gave unbiased estimates for *B* up to 10, but overestimates for *B* of 20. This is because, for high *B*, many replicates had inbreeding classes with zero survivors, which have to be excluded from calculations using Morton et al.'s (1956) model. This affected 2,552 out of 10,000 replicates for *A *= 0.25 and *B *= 20 and 4,938 replicates for *A *= 0.75 and *B *= 20, but only 51 replicates for *A *= 0.75 and *B *= 10.

Meanwhile, GLMs with a logit link function overestimated *B* (Figure 1), particularly for higher values of *B*. Furthermore, estimates of *B* differed for different levels of *A* (i.e., differing survival rate of outbred individuals) even if *B* remained unchanged. Such an effect of *A* on estimates of *B* is undesirable and demonstrates that using a logit link does not provide estimates of inbreeding load that are comparable across different populations with different environmental effects on survival.

In contrast, logarithmic GLMs and maximum‐likelihood estimation consistently provided unbiased estimates of inbreeding load (Figure 1). However, maximum‐likelihood estimation of the exponential equation failed in 106 out of 80,000 simulated data sets, and its implementation in some software packages may be considered more complicated, particularly given multiple covariates. We consequently recommend using the slope of a GLM with logarithmic link function and Poisson‐distributed errors to estimate inbreeding load and to use a sandwich estimator to get appropriate confidence intervals (Zou, 2004; Supporting Information 1).

### 3.2 Comparison of effects of metrics of *F* on estimates of inbreeding load

As expected, the distributions of the four metrics of *F* differed somewhat across the focal simulated individuals. *F*_ped_ and *F*_ROH_ had only positive values, with *F*_ped_ showing a narrower range than *F*_ROH_, whereas *F*_H_ and *F*_alt_ contained both positive and negative values and thus had a wider range and a mean close to 0 (Table 3 and Figure S5 in Supporting Information 2). We also noted that values of *F*_alt_ in immigrants and their descendants were too high because *F*_alt_ strongly weighs rare alleles brought in by immigrants (see Supporting Information 2).

**Figure 2:**
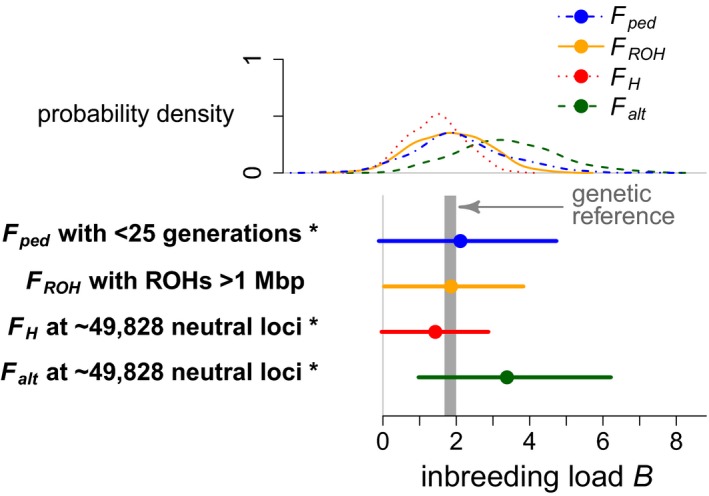
Inbreeding load estimated in a Poisson GLM with logarithmic link function and various metrics of inbreeding coefficient *F* (see main text and Table 3 for details). Curves on top of the panel show probability densities of inbreeding load estimates across all 280 analysed demes. Horizontal lines in the lower part of the panel show the 2.5% to 97.5% quantiles, and dots indicate mean estimates across all 280 demes. Asterisks (*) indicate that the mean estimate was different from the true value of inbreeding load with a *p*‐value of <5%. *F*_ped_ (blue) was based on up to 25 ancestral generations. *F*_ROH_ (orange) was based on runs of homozygosity of at least 1 Mbp. *F*_H_ (red) and *F*_alt_ (green) were calculated using all polymorphic neutral loci. The grey area (genetic reference) spans from the 2.5% quantile (1.68 lethal equivalents) to the 97.5% quantile (1.99 lethal equivalents) of actual inbreeding load calculated from the observed allele frequencies and selection coefficients at deleterious loci using equation 1

We ran genetically explicit simulations where survival was determined by genotypes at loci with deleterious alleles, and we used neutral loci or the pedigree to calculate four different metrics of *F*. The resulting estimates of inbreeding load did not yield identical results. Specifically, *F*_ped_ led to slight overestimates of inbreeding load, and moreover the variation among estimates from the replicate demes was large, making this a relatively imprecise method (Figure 2). Consequently, root mean square error (RMSE) was rather large at 1.33. *F*_ROH_ with runs of homozygosity longer than 1 Mbp provided unbiased estimates of inbreeding load, and variation in estimates was smaller than for *F*_ped_, giving an RMSE of 1.01 (Figure 2). *F*_H_ led to underestimation of inbreeding load with an RMSE of 0.86, while *F*_alt_ led to overestimation of inbreeding load with an RMSE of 2.05 (Figure 2). The 95% confidence intervals calculated for *F*_ROH_ had the best coverage probabilities, containing the true inbreeding load in 93.9% of all replicates, whereas this value was 93.6% for *F*_ped_, 90.7% for *F*_H_ and 79.6% for *F*_alt_.

Our additional analyses in Supporting Information 2 showed that results for *F*_H_ and *F*_alt_ changed only little when based on fewer genetic loci, particularly given 10,000 or more polymorphic loci (Figure S6 in Supporting Information 2). Similarly, using a shorter or longer minimum length for runs of homozygosity had little effect on estimates of inbreeding load calculated using *F*_ROH_ (Figure S6 in Supporting Information 2).

## 4 DISCUSSION

### 4.1 Comparison of statistical models to estimate inbreeding load

The concept of “inbreeding load” (Morton et al., 1956) provides a standardized and theoretically rigorous measure of the magnitude of inbreeding depression that can be compared among traits, environments and populations. While multiple statistical models (Table 2) have been used to estimate inbreeding load, our simulations show that only logarithmic models yield unbiased estimates. Specifically, a Poisson generalized linear model (GLM) with logarithmic link function, and the maximum‐likelihood exponential equation model proposed by Kalinowski and Hedrick (1998), returned unbiased estimates of inbreeding load. Other statistical models might be useful to study aspects of inbreeding other than quantification of inbreeding load.

Of these two models, the GLM with logarithmic link function is generally easy to implement. While it is not usual to model binary traits (such as survival) with Poisson error distributions and associated logarithmic links, such models return unbiased point estimates and appropriate confidence intervals can be computed (Zou, 2004; Supporting Information 1). GLMs designed to estimate inbreeding load in other traits could use error distributions other than Poisson, but using a logarithmic link function is crucial to preserve the population genetic interpretation of inbreeding load.

Meanwhile, Morton et al.'s (1956) original logarithmic regression model returned slightly biased estimates only for very high inbreeding loads (*B* = 20). Since most values of *B* estimated for survival in wild populations to date are lower than 20 (Table 1), Morton et al.'s (1956) model may, in practice, often suffice, as long as enough data are available to reliably estimate mean fitness per level of *F*. In contrast, non‐logarithmic models, in particular GLMs with logit link functions, violate key underlying population genetic assumptions and hence return estimates of the inbreeding load that are quantitatively, and conceptually, different. García‐Dorado et al. (2016) and López‐Cortegano, Bersabé, Wang, and García‐Dorado (2018) also show that logistic models are not ideal for predicting fitness under inbreeding and purging. Furthermore, GLMs with logit link functions yield different estimates of *B* depending on the arbitrary methodological choice of inbreeding levels for which model predictions are made (Supporting Information 1) and depending on the survival rate of outbred individuals (Figure 1). Such differences in baseline survival rate occur, for example, due to environmental differences between years or study sites.

To illustrate the problem, we used published data from Chatham Island black robins (*Petroica traversi*) (Kennedy, Grueber, Duncan, & Jamieson, 2014) to demonstrate how a logit link model can lead to erroneous comparative assessments of inbreeding load. A standard GLM with binomial errors and logit link generates estimates of inbreeding load that differ more than threefold among three focal study sites (R code in Supporting Information). Such highly different estimates emerge even though the same model provided no statistical support for the hypothesis that inbreeding load varied among sites (i.e., the site‐by‐*F* interaction was not significant and excluded from the model). This major apparent discrepancy in interpretation arises because the survival rates of outbred individuals varied markedly among study sites, which most likely reflects ecology (Kennedy et al., 2014). Using a GLM with logarithmic link instead does not lead to such inconsistent results. Thus, predictions from models with logit links should not be used to estimate inbreeding load. A number of estimates of “lethal equivalents” in the literature, particularly in more recent literature, are not in fact equivalent and cannot be meaningfully quantitatively compared.

### 4.2 Comparison of effects of metrics of *F* on estimates of inbreeding load

Our genetically explicit genomic simulations showed that fitting the same (appropriate) statistical model using different metrics of *F* (Table 3) returned quantitatively different estimates of the inbreeding load. Of three metrics derived from genetic markers, only that based on runs of homozygosity (*F*_ROH_) provided unbiased estimates. *F*_H_ systematically underestimated inbreeding load, but showed the lowest RMSE. Meanwhile, *F*_ped_ slightly and *F*_alt_ considerably overestimated inbreeding load. Our additional analyses of subsets of individuals and loci imply that if much larger data sets were available, estimates based on *F*_H_, *F*_ped_ and *F*_alt_ would likely still be biased whereas estimates based on *F*_ROH_ would not, while the RMSE for *F*_ROH_ would likely decrease (Supporting Information 2). Given appropriate genomic data, *F*_ROH_ may therefore be the best metric of inbreeding for quantification of inbreeding load.

Yengo et al. (2017) concluded from simulations that *F*_H_ and particularly *F*_alt_ were the best metrics to quantify inbreeding depression. However, they simulated trait values as a function of an inbreeding coefficient that was calculated in a similar way as *F*_H_ and *F*_alt_, not based on genetically explicit simulations. This shortcut is likely to bias results in favour of metrics with similar properties, leading to conclusions that simply reflect simulation methodology (Kardos et al., 2018). Our genetically explicit simulations, where both trait values and inbreeding coefficients are emergent properties of Mendelian inheritance, genetic drift and selection, show that *F*_ROH_ yields less biased estimates of the inbreeding load than *F*_H_ and *F*_alt_ (see also Keller et al., 2011).

Although *F*_ped_ has similar properties to *F*_ROH_, it yields slight overestimates of the inbreeding load. Pedigrees measure *expected* identity by descent and not variation due to Mendelian sampling and recombination, whereas a large number of genetic markers allow measuring variation in *realized* identity by descent (Franklin, 1977; Hill & Weir, 2011; Leutenegger et al., 2003; Stam, 1980). High‐density marker‐based metrics of inbreeding consequently showed higher correlations with genome‐wide identity by descent than *F*_ped_ in simulation studies (Kardos, Luikart, & Allendorf, 2015; Keller et al., 2011; Wang, 2016), as is expected when realized identity by descent randomly deviates from its expectation based on *F*_ped_. In general, independent random errors in the independent variable (i.e., *F*) increase the variance and may lead to biased regression slopes (Carroll, Ruppert, Stefanski, & Crainiceanu, 2006; Reid et al., 2014). Overestimation, such as we observed, might arise if *F*_ped_ systematically underestimates genomic inbreeding, for example due to selection and resulting reduced variance (Groen, Kennedy, & Eissen, 1995). Indeed, simulations by Curik, Sölkner, and Stipic (2001) showed that regression slopes of trait values on *F* were overestimated when using *F*_ped_ instead of realized genomic inbreeding, because *F*_ped_ underestimated the variance in identity by descent. Although desirable and increasingly feasible (Kardos et al., 2016), generating genomic data to measure inbreeding is not without challenges and may not be an option for every research programme (Andrews, Good, Miller, Luikart, & Hohenlohe, 2016; Shafer et al., 2017; Sims, Sudbery, Ilott, Heger, & Ponting, 2014). In these cases, pedigrees of sufficient depth will yield reasonable if slightly biased estimates of inbreeding load. However, if an assembled reference genome of sufficient quality and a dense genetic marker data set are available, we recommend using *F*_ROH_ and as many individuals as possible for estimation of inbreeding load.

### 4.3 Implications for wild populations

Our results show that estimates of inbreeding load are contingent on the underlying statistical model and the metric of *F*, implying that diverse published estimates are often not equivalent and impeding quantitative comparison. We thus collated published estimates of inbreeding loads in wild vertebrate populations that used unbiased methods in Table 1 and explain in the R code in the Supporting Information why other estimates were deemed to not be comparable. Not all studies of inbreeding depression reported estimates of inbreeding load, but they sometimes contained sufficient data to allow approximate calculation (details of analyses and exclusions, and R code, are in the Supporting Information). We mainly attempted to recalculate estimates of inbreeding load calculated in review studies by O'Grady et al. (2006) and Frankham et al. (2017, table 3.2). We describe the detailed methods in the R code in the Supporting Information and also explain there why some values differ. We did not list some of the previously reported estimates, mainly because they were not from wild populations or for various issues that we explain in the R code in the Supporting Information. For example, the highest value among vertebrate populations cited by Frankham et al. (2017) is based on a study on red deer (*Cervus elaphus*) (Huisman et al., 2016) that did not report inbreeding load and that used logit links and *F*_alt_ to analyse inbreeding depression. As we have shown here, such estimates of inbreeding load may be unreliable. The same concerns apply to the even higher estimates of inbreeding load reported for the same study of red deer by Hedrick and García‐Dorado (2016).

When using only estimates from models known to have little bias, a mean inbreeding load for survival until sexual maturity of 3.5 haploid lethal equivalents was found among wild vertebrate populations (Table 1). This value is higher than the mean of 2.3 reported for mammals in captivity (Ralls et al., 1988). We did not observe a recent increase in reported inbreeding load estimates from the wild as previously noted (Hedrick & García‐Dorado, 2016). However, there are not many reliable estimates of inbreeding load available for wild vertebrate populations and especially not for measures of lifetime fitness. To improve this situation, we encourage researchers to explicitly calculate and report inbreeding load for their study populations whenever possible. Furthermore, study systems where lifetime reproductive success is well known offer interesting prospects for quantification of inbreeding load in measures of total fitness. The widespread availability of genomic methods will ease the challenge of measuring inbreeding in wild animals and plants in the coming years. However, the difficulty of accurately measuring fitness in wild populations will remain. Thus, detailed long‐term study populations where survival and reproduction can be monitored in detail will become increasingly valuable for ecological and evolutionary genomics.

### 4.4 Limitations

Although our recommendation to use *F*_ROH_ for measuring inbreeding is in line with other studies (e.g., Keller et al., 2011), there are limitations to our simulations and hence quantitative conclusions. We investigated the performance of different metrics of *F* given a metapopulation of 30 demes of fixed size connected by little dispersal and gene flow. Quantitative conclusions will likely change given different structures and resulting means and variances in *F*. Indeed, *F*_H_ may perform well under some demographic scenarios (Figure S13 in Supporting Information 4). Extensive further studies on the effects of different demographic scenarios on mean and variance of metrics of *F* and their usefulness to measure inbreeding load are desirable. So far, several demographic scenarios support our main conclusion that *F*_ROH_ is the least biased metric to estimate inbreeding load (Supporting Information 4). However, our simulations were conducted using a metapopulation at near‐equilibrium of genetic drift, migration, mutation and selection. Non‐equilibrium conditions created by recent reductions in population size may lead to overestimates of inbreeding load when using *F*_ped_ and when not accounting for purging (García‐Dorado et al., 2016; López‐Cortegano et al., 2018). Other research questions may not focus on the inbreeding load but on correlations between *F* and fitness measures. Then, a different statistical model and a different metric of *F* may perform better. For example in our simulations, *F*_alt_ yielded the strongest correlation with survival (Figure S12 in Supporting Information 4).

Neither an appropriate metric of *F*, nor an appropriate statistical model, can guarantee an unbiased estimate of inbreeding load if other assumptions of the underlying theory are violated. In particular, if the assumption of independent effects of loci is violated, for example due to epistasis or additive rather than multiplicative effects *among* loci, different statistical procedures may be required. If inbreeding depression is mainly due to overdominance rather than partial directional dominance, biases in estimates of inbreeding load may also change (Curik et al., 2001). Similarly, further research is needed to assess what would change if inbreeding depression was mainly caused by few loci with large effects, such as recessive lethal mutations. Further bias in estimates of inbreeding load could arise if there are nonrandom associations between individual *F* values and environmental quality, if propensity to inbreed is correlated with fitness‐related heritable traits (Becker, Hegelbach, Keller, & Postma, 2016; Reid, Arcese, & Keller, 2008), or if parental investment differs depending on offspring *F* (Duthie, Lee, & Reid, 2016). In such cases, use of the metrics and models that we have highlighted may need to be coupled with experiments that break associations between *F* and environmental and parental effects, or with more sophisticated regression models that additionally account for additive genetic effects.

## DATA ARCHIVING STATEMENT

Code for computer simulations is uploaded as Supporting Information.

## References

[eva12713-bib-0001] Andrews, K. R. , Good, J. M. , Miller, M. R. , Luikart, G. , & Hohenlohe, P. A. (2016). Harnessing the power of RADseq for ecological and evolutionary genomics. Nature Reviews Genetics, 17, 81–92. 10.1038/nrg.2015.28 PMC4823021 26729255

[eva12713-bib-0002] Armbruster, P. , & Reed, D. H. (2005). Inbreeding depression in benign and stressful environments. Heredity, 95, 235–242. 10.1038/sj.hdy.6800721 16077737

[eva12713-bib-0003] Armstrong, D. P. , & Cassey, P. (2007). Estimating the effect of inbreeding on survival. Animal Conservation, 10, 487–492. 10.1111/j.1469-1795.2007.00139.x

[eva12713-bib-0004] Becker, P. J. J. , Hegelbach, J. , Keller, L. F. , & Postma, E. (2016). Phenotype‐associated inbreeding biases estimates of inbreeding depression in a wild bird population. Journal of Evolutionary Biology, 29, 35–46. 10.1111/jeb.12759 26362803

[eva12713-bib-0005] Bérénos, C. , Ellis, P. A. , Pilkington, J. G. , & Pemberton, J. M. (2016). Genomic analysis reveals depression due to both individual and maternal inbreeding in a free‐living mammal population. Molecular Ecology, 25, 3152–3168. 10.1111/mec.13681 27135155 PMC4950049

[eva12713-bib-0006] Brown, J. L. , & Brown, E. R. (1998). Are inbred offspring less fit? Survival in a natural population of Mexican jays. Behavioral Ecology, 9, 60–63. 10.1093/beheco/9.1.60

[eva12713-bib-0007] Caballero, A. , Bravo, I. , & Wang, J. (2017a). Inbreeding load and purging: Implications for the short‐term survival and the conservation management of small populations. Heredity, 118, 177–185. 10.1038/hdy.2016.80 27624114 PMC5234482

[eva12713-bib-0008] Caballero, A. , Bravo, I. , & Wang, J. (2017b). The risk of forcing inbreeding in conservation programmes: A reply to Theodorou and Couvet. Heredity, 19, 51–53. 10.1038/hdy.2017.17 PMC5520138 28327579

[eva12713-bib-0009] Carroll, R. J. , Ruppert, D. , Stefanski, L. A. , & Crainiceanu, C. M. (2006). Measurement error in nonlinear models: A modern perspective, 2nd ed. Boca Raton, FL: Chapman & Hall/CRC 10.1201/CHMONSTAAPP

[eva12713-bib-0010] Charlesworth, D. , & Charlesworth, B. (1987). Inbreeding depression and its evolutionary consequences. Annual Review of Ecology and Systematics, 18, 237–268. 10.1146/annurev.es.18.110187.001321

[eva12713-bib-0011] Charlesworth, D. , & Willis, J. H. (2009). The genetics of inbreeding depression. Nature Reviews Genetics, 10, 783–796. 10.1038/nrg2664 19834483

[eva12713-bib-0012] Crow, J. F. , & Kimura, M. (1970). An introduction to population genetics theory. Caldwell, NJ: The Blackburn Press.

[eva12713-bib-0013] Curik, I. , Ferenčaković, M. , & Sölkner, J. (2014). Inbreeding and runs of homozygosity: A possible solution to an old problem. Livestock Science, 166, 26–34. 10.1016/j.livsci.2014.05.034

[eva12713-bib-0014] Curik, I. , Sölkner, J. , & Stipic, N. (2001). The influence of selection and epistasis on inbreeding depression estimates. Journal of Animal Breeding and Genetics, 118, 247–262. 10.1046/j.1439-0388.2001.00284.x

[eva12713-bib-0015] Dietz, J. M. , Baker, A. J. , & Ballou, J. D. (2000). Demographic evidence of inbreeding depression in wild golden lion tamarins In Young A. G., & Clarke G. M. (Eds.), Genetics, demography and viability of fragmented populations (pp. 203–211). Cambridge, UK: Cambridge University Press 10.1017/CBO9780511623448

[eva12713-bib-0016] Duthie, A. B. , Lee, A. M. , & Reid, J. M. (2016). Inbreeding parents should invest more resources in fewer offspring. Proceedings of The Royal Society B: Biological Sciences, 283, 20161845 10.1098/rspb.2016.1845 PMC5136589 27881747

[eva12713-bib-0018] Fisher, R. A. (1954). A fuller theory of “junctions” in inbreeding. Heredity, 8, 187–197. 10.1038/hdy.1954.17

[eva12713-bib-0019] Fox, C. W. , & Reed, D. H. (2010). Inbreeding depression increases with environmental stress: An experimental study and meta‐analysis. Evolution, 65, 246–258. 20731715 10.1111/j.1558-5646.2010.01108.x

[eva12713-bib-0020] Frankham, R. , Ballou, J. D. , Ralls, K. , Eldridge, M. D. B. , Dudash, M. R. , Fenster, C. B. , … Sunnucks, P. (2017). Genetic management of fragmented animal and plant populations. Oxford, UK: Oxford University Press 10.1093/oso/9780198783398.001.0001

[eva12713-bib-0021] Franklin, I. R. (1977). The distribution of the proportion of the genome which is homozygous by descent in inbred individuals. Theoretical Population Biology, 11, 60–80. 10.1016/0040-5809(77)90007-7 404725

[eva12713-bib-0022] Fredrickson, R. J. , Siminski, P. , Woolf, M. , & Hedrick, P. W. (2007). Genetic rescue and inbreeding depression in Mexican wolves. Proceedings of the Royal Society B, 274, 2365–2371. 10.1098/rspb.2007.0785 17609180 PMC2288557

[eva12713-bib-0023] García‐Dorado, A. , Wang, J. , & López‐Cortegano, E. (2016). Predictive model and software for inbreeding‐purging analysis of pedigreed populations. G3 Genes – Genomes – Genetics 6:3593–3601. 27605515 10.1534/g3.116.032425 PMC5100858

[eva12713-bib-0024] Germain, R. R. , Arcese, P. , & Reid, J. M. (2018). The consequences of polyandry for sibship structures, distributions of relationships and relatedness, and potential for inbreeding in a wild population. American Naturalist, 191, 638–657. 10.1086/696855 29693437

[eva12713-bib-0025] Glémin, S. , Vimond, L. , Ronfort, J. , Bataillon, T. , & Mignot, A. (2006). Marker‐based investigation of inbreeding depression in the endangered species *Brassica insularis* . Heredity, 97, 304–311. 10.1038/sj.hdy.6800870 16850037

[eva12713-bib-0026] Grant, P. R. , Grant, B. R. , & Petren, K. (2001). A population founded by a single pair of individuals: Establishment, expansion, and evolution. Genetica, 112–113, 359–382. 10.1023/A:1013363032724 11838776

[eva12713-bib-0027] Groen, A. F. , Kennedy, B. W. , & Eissen, J. J. (1995). Potential bias in inbreeding depression estimates when using pedigree relationships to assess the degree of homozygosity for loci under selection. Theoretical and Applied Genetics, 91, 665–671. 24169896 10.1007/BF00223295

[eva12713-bib-0028] Grueber, C. E. , Nakagawa, S. , Laws, R. J. , & Jamieson, I. G. (2011). Multimodel inference in ecology and evolution: Challenges and solutions. Journal of Evolutionary Biology, 24, 699–711. 10.1111/j.1420-9101.2010.02210.x 21272107

[eva12713-bib-0029] Guillaume, F. , & Rougemont, J. (2006). Nemo: An evolutionary and population genetics programming framework. Bioinformatics, 22, 2556–2557. 10.1093/bioinformatics/btl415 16882649

[eva12713-bib-0030] Hedrick, P. W. , & García‐Dorado, A. (2016). Understanding inbreeding depression, purging, and genetic rescue. Trends in Ecology & Evolution, 31, 940–952. 10.1016/j.tree.2016.09.005 27743611

[eva12713-bib-0031] Hedrick, P. W. , Hellsten, U. , & Grattapaglia, D. (2016). Examining the cause of high inbreeding depression: Analysis of whole‐genome sequence data in 28 selfed progeny of Eucalyptus grandis. New Phytologist, 209, 600–611. 10.1111/nph.13639 26356869

[eva12713-bib-0032] Hedrick, P. W. , & Kalinowski, S. T. (2000). Inbreeding depression in conservation biology. Annual Review of Ecology and Systematics, 31, 139–162. 10.1146/annurev.ecolsys.31.1.139

[eva12713-bib-0033] Hill, W. G. , & Weir, B. S. (2011). Variation in actual relationship as a consequence of Mendelian sampling and linkage. Genetics Research, 93, 47–64. 10.1017/S0016672310000480 21226974 PMC3070763

[eva12713-bib-0034] Hoeck, P. E. A. , Wolak, M. E. , Switzer, R. A. , Kuehler, C. M. , & Lieberman, A. A. (2015). Effects of inbreeding and parental incubation on captive breeding success in Hawaiian crows. Biological Conservation, 184, 357–364. 10.1016/j.biocon.2015.02.011

[eva12713-bib-0035] Hoffman, J. I. , Simpson, F. , David, P. , Rijks, J. M. , Kuiken, T. , Thorne, M. A. S. , … Dasmahapatra, K. K. (2014). High‐throughput sequencing reveals inbreeding depression in a natural population. Proceedings of the National Academy of Sciences of the United States of America, 111, 3775–3780. 10.1073/pnas.1318945111 24586051 PMC3956162

[eva12713-bib-0036] Howrigan, D. P. , Simonson, M. A. , & Keller, M. C. (2011). Detecting autozygosity through runs of homozygosity: A comparison of three autozygosity detection algorithms. BMC Genomics, 12, 460 10.1186/1471-2164-12-460 21943305 PMC3188534

[eva12713-bib-0037] Huisman, J. , Kruuk, L. E. B. , Ellis, P. A. , Clutton‐Brock, T. , & Pemberton, J. M. (2016). Inbreeding depression across the lifespan in a wild mammal population. Proceedings of the National Academy of Sciences, 113, 3585–3590. 10.1073/pnas.1518046113 PMC4822623 26979959

[eva12713-bib-0038] Jamieson, I. G. , Tracy, L. N. , Fletcher, D. , & Armstrong, D. P. (2007). Moderate inbreeding depression in a reintroduced population of North Island robins. Animal Conservation, 10, 95–102. 10.1111/j.1469-1795.2006.00078.x

[eva12713-bib-0039] Jimenez, J. A. , Hughes, K. A. , Alaks, G. , Graham, L. , & Lacy, R. C. (1994). An experimental study of inbreeding depression in a natural habitat. Science, 266, 271–273. 10.1126/science.7939661 7939661

[eva12713-bib-0040] Kalinowski, S. T. , & Hedrick, P. W. (1998). An improved method for estimating inbreeding depression in pedigrees. Zoo Biology, 17, 481–497. 10.1002/(ISSN)1098-2361

[eva12713-bib-0041] Kardos, M. , Luikart, G. , & Allendorf, F. W. (2015). Measuring individual inbreeding in the age of genomics: Marker‐based measures are better than pedigrees. Heredity, 115, 63–72. 10.1038/hdy.2015.17 26059970 PMC4815495

[eva12713-bib-0042] Kardos, M. , Nietlisbach, P. , & Hedrick, P. W. (2018). How should we compare different genomic estimates of the strength of inbreeding depression? Proceedings of the National Academy of Sciences, 115, E2492–E2493. 10.1073/pnas.1714475115 PMC5856524 29467294

[eva12713-bib-0043] Kardos, M. , Taylor, H. R. , Ellegren, H. , Luikart, G. , & Allendorf, F. W. (2016). Genomics advances the study of inbreeding depression in the wild. Evolutionary Applications, 9, 1205–1218. 10.1111/eva.12414 27877200 PMC5108213

[eva12713-bib-0044] Keller, L. F. (1998). Inbreeding and its fitness effects in an insular population of song sparrows (*Melospiza melodia*). Evolution, 52, 240–250. 28568167 10.1111/j.1558-5646.1998.tb05157.x

[eva12713-bib-0045] Keller, L. F. , Grant, P. R. , Grant, B. R. , & Petren, K. (2002). Environmental conditions affect the magnitude of inbreeding depression in survival of Darwin's finches. Evolution, 56, 1229–1239. 10.1111/j.0014-3820.2002.tb01434.x 12144022

[eva12713-bib-0046] Keller, M. C. , Visscher, P. M. , & Goddard, M. E. (2011). Quantification of inbreeding due to distant ancestors and its detection using dense single nucleotide polymorphism data. Genetics, 189, 237–249. 10.1534/genetics.111.130922 21705750 PMC3176119

[eva12713-bib-0047] Keller, L. F. , & Waller, D. M. (2002). Inbreeding effects in wild populations. Trends in Ecology & Evolution, 17, 230–241. 10.1016/S0169-5347(02)02489-8

[eva12713-bib-0048] Kennedy, E. S. , Grueber, C. E. , Duncan, R. P. , & Jamieson, I. G. (2014). Severe inbreeding depression and no evidence of purging in an extremely inbred wild species‐the Chatham Island black robin. Evolution, 68, 987–995. 10.1111/evo.12315 24303793

[eva12713-bib-0049] Knief, U. , Hemmrich‐Stanisak, G. , Wittig, M. , Franke, A. , Griffith, S. C. , Kempenaers, B. , & Forstmeier, W. (2015). Quantifying realized inbreeding in wild and captive animal populations. Heredity, 114, 397–403. 10.1038/hdy.2014.116 25585923 PMC4359978

[eva12713-bib-0050] Knief, U. , Kempenaers, B. , & Forstmeier, W. (2017). Meiotic recombination shapes precision of pedigree‐ and marker‐based estimates of inbreeding. Heredity, 118, 239–248. 10.1038/hdy.2016.95 27804967 PMC5315531

[eva12713-bib-0051] Kokko, H. , & Ots, I. (2006). When not to avoid inbreeding. Evolution, 60, 467–475. 10.1111/j.0014-3820.2006.tb01128.x 16637492

[eva12713-bib-0052] Kristensen, T. N. , & Sorensen, A. C. (2005). Inbreeding – lessons from animal breeding, evolutionary biology and conservation genetics. Animal Science, 80, 121–133.

[eva12713-bib-0053] Kruuk, L. E. B. , Sheldon, B. C. , & Merilä, J. (2002). Severe inbreeding depression in collared flycatchers (*Ficedula albicollis*). Proceedings of the Royal Society B, 269, 1581–1589. 10.1098/rspb.2002.2049 12184828 PMC1691074

[eva12713-bib-0054] Lacy, R. C. (1997). Importance of genetic variation to the viability of mammalian populations. Journal of Mammalogy, 78, 320–335. 10.2307/1382885

[eva12713-bib-0055] Laine, V. N. , Gossmann, T. I. , Schachtschneider, K. M. , Garroway, C. J. , Madsen, O. , Verhoeven, K. J. F. , … Groenen, M. A. (2016). Evolutionary signals of selection on cognition from the great tit genome and methylome. Nature Communications, 7, 10474 10.1038/ncomms10474 PMC4737754 26805030

[eva12713-bib-0056] Lascoux, M. , & Lee, J. K. (1998). One step beyond lethal equivalents: Characterization of deleterious loci in the rapid cycling *Brassica rapa* L. base population. Genetica, 104, 161–170. 10.1023/A:1003441713325 16220375

[eva12713-bib-0057] Lee, J. K. , Lascoux, M. , & Nordheim, E. V. (1996). Number of lethal equivalents in human populations: How good are the previous estimates? Heredity, 77, 209–216. 10.1038/hdy.1996.126 8760402

[eva12713-bib-0058] Leroy, G. (2014). Inbreeding depression in livestock species: Review and meta‐analysis. Animal Genetics, 45, 618–628. 10.1111/age.12178 24975026

[eva12713-bib-0059] Leutenegger, A. L. , Prum, B. , Genin, E. , Verny, C. , Lemainque, A. , Clerget‐Darpoux, F. , & Thompson, E. A. (2003). Estimation of the inbreeding coefficient through use of genomic data. American Journal of Human Genetics, 73, 516–523. 10.1086/378207 12900793 PMC1180677

[eva12713-bib-0060] Liberg, O. , Andrén, H. , Pedersen, H.‐C. , Sand, H. , Sejberg, D. , Wabakken, P. , … Bensch, S. (2005). Severe inbreeding depression in a wild wolf (*Canis lupus*) population. Biology Letters, 1, 17–20. 10.1098/rsbl.2004.0266 17148117 PMC1629062

[eva12713-bib-0061] López‐Cortegano, E. , Bersabé, D. , Wang, J. , & García‐Dorado, A. (2018). Detection of genetic purging and predictive value of purging parameters estimated in pedigreed populations. Heredity, 121, 38–51. 10.1038/s41437-017-0045-y 29434337 PMC5997712

[eva12713-bib-0062] Lynch, M. , & Walsh, B. (1998). Genetics and analysis of quantitative traits. Sunderland, MA: Sinauer Associates.

[eva12713-bib-0063] Makov, E. , & Bittles, A. H. (1986). On the choice of mathematical models for the estimation of lethal gene equivalents in man. Heredity, 57, 377–380. 10.1038/hdy.1986.136 3804766

[eva12713-bib-0064] McQuillan, R. , Leutenegger, A.‐L. , Abdel‐Rahman, R. , Franklin, C. S. , Pericic, M. , Barac‐Lauc, L. , … Wilson, J. F. (2008). Runs of homozygosity in European populations. The American Journal of Human Genetics, 83, 359–372. 10.1016/j.ajhg.2008.08.007 18760389 PMC2556426

[eva12713-bib-0065] McRae, S. B. (1996). Family values: Costs and benefits of communal nesting in the moorhen. Animal Behaviour, 52, 225–245. 10.1006/anbe.1996.0169

[eva12713-bib-0066] Morton, N. E. , Crow, J. F. , & Muller, H. J. (1956). An estimate of the mutational damage in man from data on consanguineous marriages. Proceedings of the National Academy of Sciences of the United States of America, 42, 855–863. 10.1073/pnas.42.11.855 16589958 PMC528351

[eva12713-bib-0067] Nietlisbach, P. , Keller, L. F. , Camenisch, G. , Arcese, P. , Reid, J. M. , & Postma, E. (2017). Pedigree‐based inbreeding coefficient explains more variation in fitness than heterozygosity at 160 microsatellites in a wild bird population. Proceedings of the Royal Society B, 284, 20162763 10.1098/rspb.2016.2763 28250184 PMC5360928

[eva12713-bib-0068] van Noordwijk, A. J. , & Scharloo, W. (1981). Inbreeding in an island population of the great tit. Evolution, 35, 674–688. 10.2307/2408240 28563132

[eva12713-bib-0069] van Oers, K. , Santure, A. W. , De Cauwer, I. , van Bers, N. E. M. , Crooijmans, R. P. M. A. , Sheldon, B. C. , … Groenen, M. A. (2014). Replicated high‐density genetic maps of two great tit populations reveal fine‐scale genomic departures from sex‐equal recombination rates. Heredity, 112, 307–316. 10.1038/hdy.2013.107 24149651 PMC3931172

[eva12713-bib-0070] O'Grady, J. J. , Brook, B. W. , Reed, D. H. , Ballou, J. D. , Tonkyn, D. W. , & Frankham, R. (2006). Realistic levels of inbreeding depression strongly affect extinction risk in wild populations. Biological Conservation, 133, 42–51. 10.1016/j.biocon.2006.05.016

[eva12713-bib-0071] Purcell, S. , Neale, B. , Todd‐Brown, K. , Thomas, L. , Ferreira, M. A. R. , Bender, D. , … Sham, P. C. (2007). PLINK: A tool set for whole‐genome association and population‐based linkage analyses. American Journal of Human Genetics, 81, 559–575. 10.1086/519795 17701901 PMC1950838

[eva12713-bib-0072] R Core Team (2015). R: A language and environment for statistical computing. Vienna, Austria: R Foundation for Statistical Computing.

[eva12713-bib-0073] Ralls, K. , Ballou, J. D. , & Templeton, A. (1988). Estimates of lethal equivalents and the cost of inbreeding in mammals. Conservation Biology, 2, 185–192. 10.1111/j.1523-1739.1988.tb00169.x

[eva12713-bib-0074] Reid, J. M. , Arcese, P. , & Keller, L. F. (2008). Individual phenotype, kinship, and the occurrence of inbreeding in song sparrows. Evolution, 62, 887–899. 10.1111/j.1558-5646.2008.00335.x 18248635

[eva12713-bib-0075] Reid, J. M. , Keller, L. F. , Marr, A. B. , Nietlisbach, P. , Sardell, R. J. , & Arcese, P. (2014). Pedigree error due to extra‐pair reproduction substantially biases estimates of inbreeding depression. Evolution, 68, 802–815. 10.1111/evo.12305 24171712

[eva12713-bib-0076] Sardell, R. J. , Keller, L. F. , Arcese, P. , Bucher, T. , & Reid, J. M. (2010). Comprehensive paternity assignment: Genotype, spatial location and social status in song sparrows, *Melospiza melodia* . Molecular Ecology, 19, 4352–4364. 10.1111/j.1365-294X.2010.04805.x 20819155

[eva12713-bib-0077] Shafer, A. B. A. , Peart, C. R. , Tusso, S. , Maayan, I. , Brelsford, A. , Wheat, C. W. , & Wolf, J. B. W. (2017). Bioinformatic processing of RAD‐seq data dramatically impacts downstream population genetic inference. Methods in Ecology and Evolution, 8, 907–917. 10.1111/2041-210X.12700

[eva12713-bib-0078] Sims, D. , Sudbery, I. , Ilott, N. E. , Heger, A. , & Ponting, C. P. (2014). Sequencing depth and coverage: Key considerations in genomic analyses. Nature Reviews Genetics, 15, 121–132. 10.1038/nrg3642 24434847

[eva12713-bib-0079] Smith, J. N. M. , Keller, L. F. , Marr, A. B. , & Arcese, P. (2006). Conservation and biology of small populations: The song sparrows of Mandarte Island. New York, NY: Oxford University Press.

[eva12713-bib-0080] Stam, P. (1980). The distribution of the fraction of the genome identical by descent in finite random mating populations. Genetical Research, 35, 131–155. 10.1017/S0016672300014002

[eva12713-bib-0081] Szulkin, M. , Garant, D. , McCleery, R. H. , & Sheldon, B. C. (2007). Inbreeding depression along a life‐history continuum in the great tit. Journal of Evolutionary Biology, 20, 1531–1543. 10.1111/j.1420-9101.2007.01325.x 17584246

[eva12713-bib-0082] Templeton, A. R. , & Read, B. (1983). The elimination of inbreeding depression in a captive herd of Speke's gazelle In Schonewald C., Chambers S. M., MacBryde B., & Thomas L. (Eds.), Genetics & Conservation: A reference for managing wild animal & plant populations (pp. 241–261). Caldwell, NJ: The Blackburn Press.

[eva12713-bib-0083] Templeton, A. R. , & Read, B. (1984). Factors eliminating inbreeding depression in a captive herd of Speke's gazelle (*Gazella spekei*). Zoo Biology, 3, 177–199. 10.1002/(ISSN)1098-2361

[eva12713-bib-0084] Theodorou, K. , & Couvet, D. (2017). Circular mating as an option for the genetic management of captive populations: Response to Caballero et al.. Heredity, 119, 49–50. 10.1038/hdy.2017.16 28327580 PMC5520137

[eva12713-bib-0085] Vazquez, A. I. , Bates, D. M. , Rosa, G. J. M. , Gianola, D. , & Weigel, K. A. (2010). Technical note: An R package for fitting generalized linear mixed models in animal breeding. Journal of Animal Science, 88, 497–504. 10.2527/jas.2009-1952 19820058

[eva12713-bib-0086] Waller, D. M. , Dole, J. , & Bersch, A. J. (2008). Effects of stress and phenotypic variation on inbreeding depression in *Brassica rapa* . Evolution, 62, 917–931. 10.1111/j.1558-5646.2008.00325.x 18208569

[eva12713-bib-0087] Walling, C. A. , Nussey, D. H. , Morris, A. , Clutton‐Brock, T. H. , Kruuk, L. E. B. , & Pemberton, J. M. (2011). Inbreeding depression in red deer calves. BMC Evolutionary Biology, 11, 318 10.1186/1471-2148-11-318 22039837 PMC3226574

[eva12713-bib-0088] Wang, J. (2014). Marker‐based estimates of relatedness and inbreeding coefficients: An assessment of current methods. Journal of Evolutionary Biology, 27, 518–530. 10.1111/jeb.12315 24444019

[eva12713-bib-0089] Wang, J. (2015). Does G_ST_ underestimate genetic differentiation from marker data? Molecular Ecology, 24, 3546–3558. 10.1111/mec.13204 25891752

[eva12713-bib-0090] Wang, J. (2016). Pedigrees or markers: Which are better in estimating relatedness and inbreeding coefficient? Theoretical Population Biology, 107, 4–13. 10.1016/j.tpb.2015.08.006 26344786

[eva12713-bib-0091] Wang, J. , Hill, W. G. , Charlesworth, D. , & Charlesworth, B. (1999). Dynamics of inbreeding depression due to deleterious mutations in small populations: Mutation parameters and inbreeding rate. Genetical Research, 74, 165–178. 10.1017/S0016672399003900 10584559

[eva12713-bib-0092] Willis, K. , & Wiese, R. J. (1997). Elimination of inbreeding depression from captive populations: speke's gazelle revisited. Zoo Biology, 16, 9–16. 10.1002/(ISSN)1098-2361

[eva12713-bib-0093] Wilson, A. G. , & Arcese, P. (2008). Influential factors for natal dispersal in an avian island metapopulation. Journal of Avian Biology, 39, 341–347. 10.1111/j.0908-8857.2008.04239.x

[eva12713-bib-0094] Wolak, M. E. , Arcese, P. , Keller, L. F. , Nietlisbach, P. , & Reid, J. M. (2018). Sex‐specific additive genetic variances and correlations for fitness in a song sparrow (*Melospiza melodia*) population subject to natural immigration and inbreeding. Evolution, in press. 10.1111/evo.13575 30101430

[eva12713-bib-0095] Wolak, M. E. , & Keller, L. F. (2014). Dominance genetic variance and inbreeding in natural populations In Charmantier A., Garant D., & Kruuk L. E. B. (Eds.), Quantitative genetics in the wild (pp. 104–127). Oxford, UK: Oxford University Press 10.1093/acprof:oso/9780199674237.001.0001

[eva12713-bib-0096] Wright, S. (1969). Evolution and the genetics of populations. Volume 2: The theory of gene frequencies. Chicago, IL: University of Chicago Press.

[eva12713-bib-0097] Wright, S. (1977). Evolution and the genetics of populations. Volume 3: Experimental results and evolutionary deductions. Chicago, IL: University of Chicago Press.

[eva12713-bib-0098] Yang, J. , Benyamin, B. , McEvoy, B. P. , Gordon, S. , Henders, A. K. , Nyholt, D. R. , … Visscher, P. M. (2010). Common SNPs explain a large proportion of the heritability for human height. Nature Genetics, 42, 565–569. 10.1038/ng.608 20562875 PMC3232052

[eva12713-bib-0099] Yang, J. , Lee, S. H. , Goddard, M. E. , & Visscher, P. M. (2011). GCTA: A tool for genome‐wide complex trait analysis. The American Journal of Human Genetics, 88, 76–82. 10.1016/j.ajhg.2010.11.011 21167468 PMC3014363

[eva12713-bib-0100] Yengo, L. , Zhu, Z. , Wray, N. R. , Weir, B. S. , Yang, J. , Robinson, M. R. , & Visscher, P. M. (2017). Detection and quantification of inbreeding depression for complex traits from SNP data. Proceedings of the National Academy of Sciences, 114, 8602–8607. 10.1073/pnas.1621096114 PMC5558994 28747529

[eva12713-bib-0104] Zou, G. (2004). A modified Poisson regression approach to prospective studies with binary data. American Journal of Epidemiology, 159, 702–706. 10.1093/aje/kwh090 15033648

