# Supplementary material for: Nonequivalent lethal equivalents: Models and inbreeding metrics for unbiased estimation of inbreeding load
Source: Evol Appl. 2018 Oct 23;12(2):266–79. doi: 10.1111/eva.12713 (PMC6346663; doi:10.1111/eva.12713)
Supplement: Supplementary file 1 [file EVA-12-266-s001.pdf]

*Supporting Information*

Pirmin Nietlisbach, Stefanie Muff, Jane M. Reid, Michael C. Whitlock, Lukas F. Keller
(2018). Nonequivalent lethal equivalents: Models and inbreeding metrics for unbiased estimation of inbreeding load. Evolutionary Applications.

# Supporting Information 1: Comparison and description of regression models with simulated genetic data

## *Methods*

The simulations presented in the main manuscript in the section "Comparison of statistical models to estimate inbreeding load" were designed to compare the performance of the statistical models summarised in Table 2 and illustrated in Figure S4. To that end, $F$ was assumed to be known precisely and to directly affect fitness, thereby allowing for unambiguous assessment of biases in the statistical models. However, Figures S2 and S3 show that random errors in inbreeding coefficients or survival probability did not affect results. In addition to these analyses presented in the main manuscript, we also used genetic data from the simulated metapopulations where survival was determined by loci with deleterious mutations (see Figure S1 for an illustration of the relationship and distribution of selection and dominance coefficients). These analyses serve as an alternative further comparison of statistical models and to illustrate and describe the statistical models in more detail. Again, we only used $F_{ROH}$ calculated from runs of homozygosity larger than 1 Mbp and excluded immigrants from the dataset. Analyses were conducted in R v3.2.3 (R Core Team 2015), and performed for each deme separately, resulting in a sample of 280 demes from a total of 10 metapopulations. The following headings refer to the names used in Table 2 to label the statistical models.

### *Morton & TR*

First, we applied the regression model proposed by Morton et al. (1956). We grouped all individuals into 10 similarly sized classes of inbreeding coefficients. For each class, $-log_e(s_c)$ was calculated, with $s_c$ being the survival rate calculated using the small sample size correction proposed by Templeton and Read (1983, 1984): $s_c = (n_{survived} + 1)/(n_{sampled} + 2)$, with $n_{sampled}$ being the number of individuals alive at the

start of the investigated period (i.e. here at virtual birth), and $n_{survived}$ being the number of individuals still alive at the end of the investigated period (i.e. here during the next breeding season). The regression of $-log_e(s_c)$ on mean $F_{ROH}$ per inbreeding class was performed by iteratively weighting the 10 data points with $n_{sampled}s_{exp}/(1-s_{exp})$, where $s_{exp}$ is the survival rate as predicted by the regression (Morton et al. 1956). $s_{exp}$ in the first iteration was set to $s_c$ and then in each successive iteration replaced with the predictions of the previous regression, until the regression slope changed by less than 0.0001 in successive iterations.

### *Morton et al.*

As shown previously (Kalinowski and Hedrick 1998), the small sample size correction proposed by Templeton and Read (1983, 1984) introduces not easily predictable biases into estimates of inbreeding load. We therefore also performed the same analysis without this correction by using the unadjusted survival rate $s_c = n_{survived}/n_{sampled}$. This procedure fails when there are no surviving individuals in an inbreeding class (because it results in $-log_e(0)$, which is not defined), in which case we would not estimate inbreeding load for such a replicate. However, this did not happen in any of the 280 demes.

### *Exponent. ML*

The second model followed Kalinowski and Hedrick (1998) and estimated $y_F = y_0 e^{-BF}$ with $y_0 = e^{-A}$ directly with maximum-likelihood. We minimized the negative log-likelihood with the R function *mle*. For illustration, we also directly calculated the log-likelihood for $y_0$ larger than 0 and smaller than 1, and for $B$ ranging from 0 to 10. Note that this model was fitted to the individual-level binary survival data (i.e. dead or alive), and was not grouped into classes of similar inbreeding coefficients.

### *GLM log-link*

As a third model, we fitted generalized linear models (GLMs) with Poisson-distributed errors and logarithmic link function, and with the binary variable of individual survival (i.e. dead or alive) as response variable and $F_{ROH}$ as explanatory variable. Glémin et al. (2006) used a similar model, but analysed survival rate of groups of individuals with similar inbreeding coefficients in a GLM with binomial error distribution (we instead used individual-level data). Using a GLM with logarithmic link function and a Poisson distribution instead of a binomial distribution usually avoids possible convergence problems and point estimates remain unbiased, but standard confidence intervals are typically too large (Zou 2004). This issue can

be resolved by using the so-called sandwich estimator, a robust error variance procedure (Zou 2004). It is important here to use a logarithmic link function, in which case a Poisson error distribution is recommended also when binary survival data are analysed (Zou 2004). Robust standard errors were calculated by fitting the Poisson GLM and then using the R command coeftest(glm.modelname, vcov=sandwich) of the R libraries *lmtest* and *sandwich* (Zeileis and Hothorn 2002; Zeileis 2004, 2006), and 95% Wald confidence intervals were estimated as the point estimate plus and minus 1.96 times the robust standard error. As an alternative, researchers could choose to use a parametric bootstrapping model instead (Efron and Tibshirani 1993). For examples see the R code in the Supporting Information.

## *GLM logit-link*

The fourth model included fitting a GLM with binomial error structure with a logit link function (Armstrong and Cassey 2007; Grueber et al. 2011). Survival rate $y_i$ was then predicted for various pairs of different inbreeding levels $F_i$, and equation 3 was applied, as recommended by Grueber et al. (2011). The predicted survival rate $y_0$ for outbred individuals was used to estimate the intercept $A = -log_e(y_0)$.

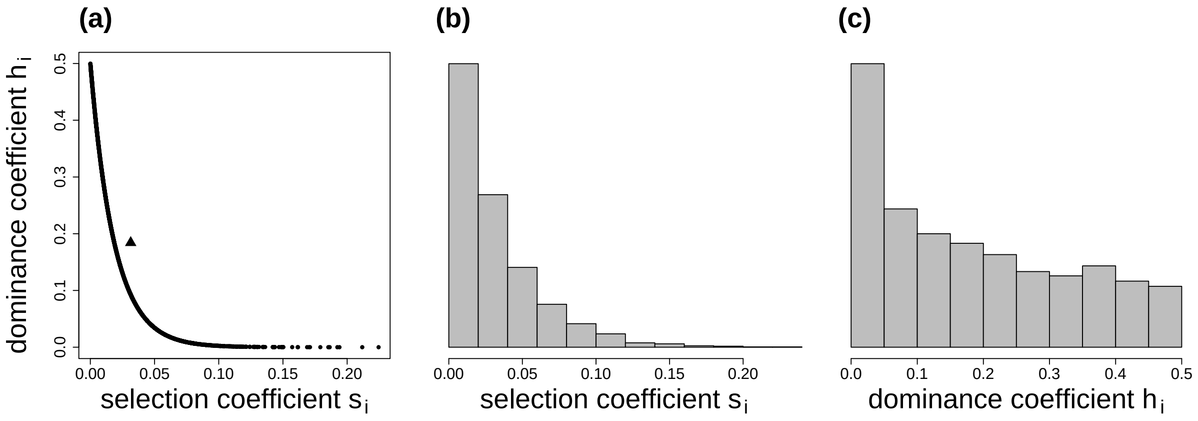


**Figure S1. (a)** The relationship is shown of the selection and dominance coefficients of the 2,500 loci with deleterious mutations simulated in Nemo. Dots represent individual loci, and the triangle represent the mean across all 2,500 loci. The distributions of values of **(b)** the selection coefficients and **(c)** the dominance coefficients are also shown.

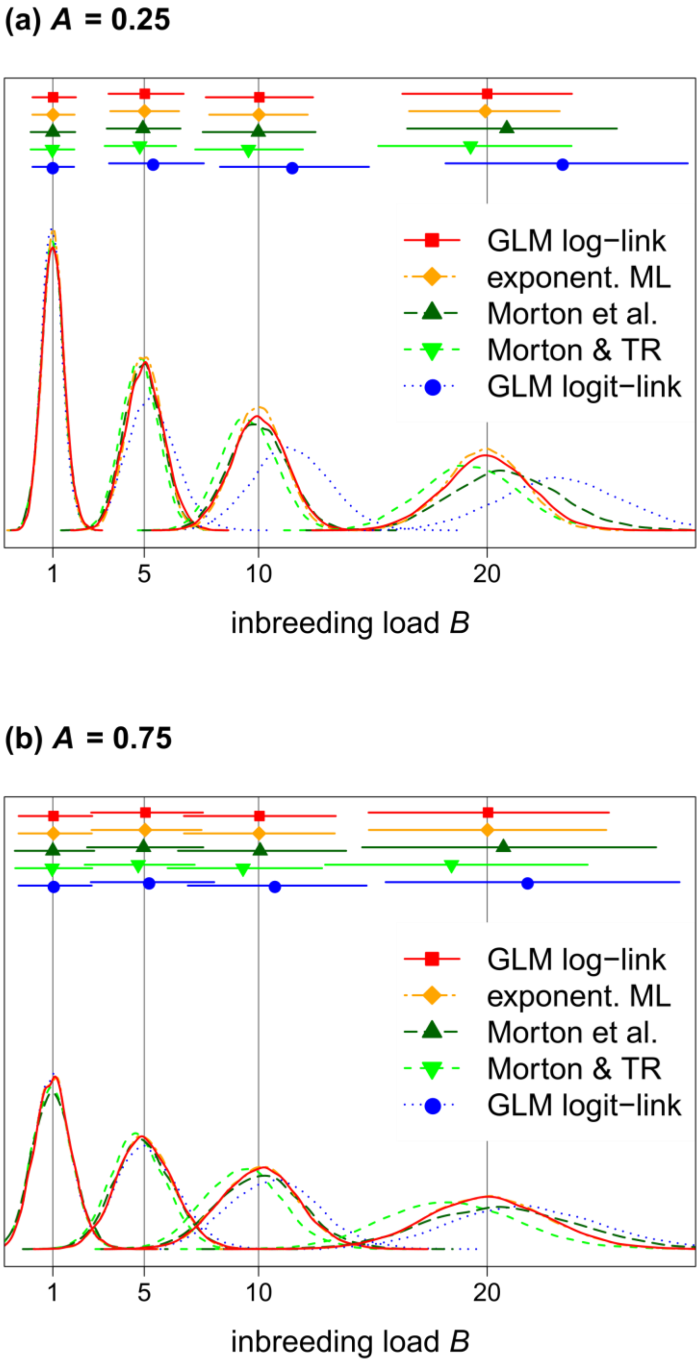


**Figure S2.** Random errors in inbreeding coefficients did not affect biases of statistical models. Here, the same simulations are shown as in Figure 1, with the exception that here inbreeding coefficients $F$ contain random errors. Thus, equation 4 was modified here to be $\pi_F = e^{-A-B(F+\sigma_F)}$, with $\sigma_F$ being a random number drawn from a normal distribution with a mean of 0 and a standard deviation of 0.01. This simple modification allowed for negative values of F, because the purpose of these simulations was just to investigate how results were affected by random variation as could arise for example from individuals with the same value of F differing in realized identity-by-descent at loci with deleterious alleles.

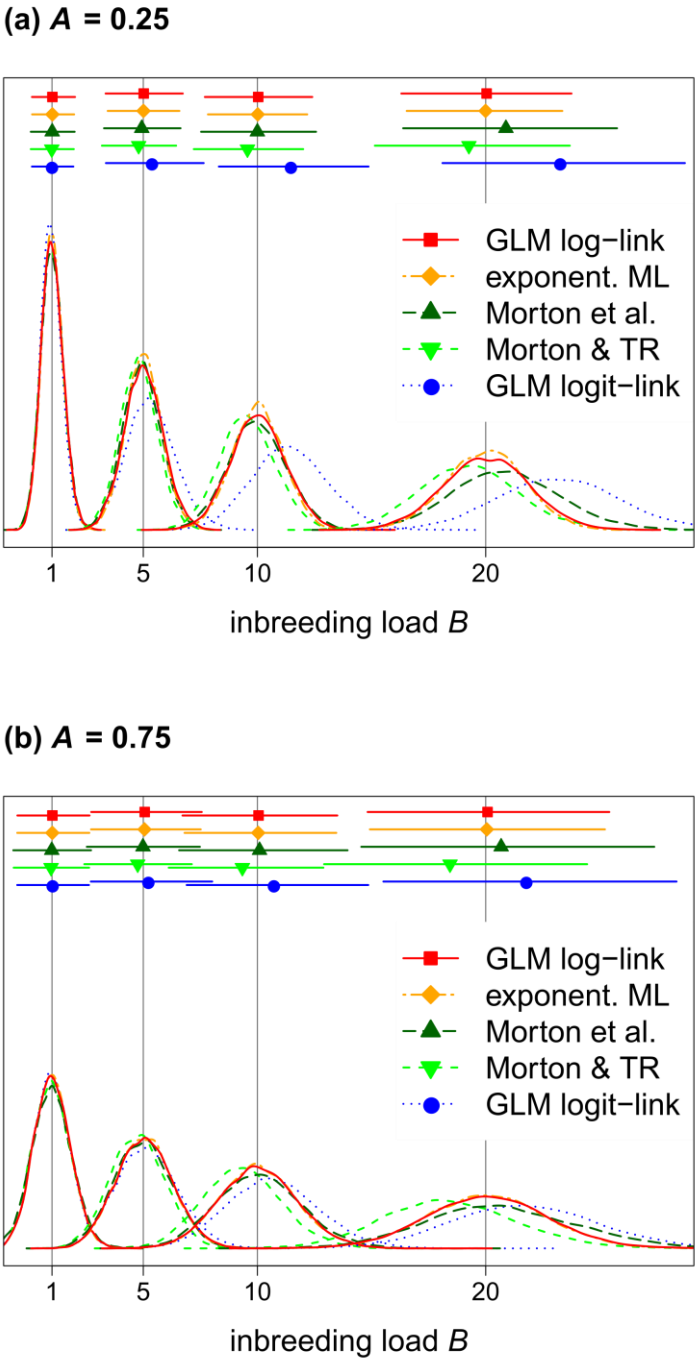


**Figure S3.** Random errors in survival probability did not affect biases of statistical models. Here, the same simulations are shown as in Figure 1, with the exception that here survival probabilities $\pi_F$ contain random errors. Thus, equation 4 was modified here to be $\pi_F = \sigma_\pi\, e^{-A-BF}$, with $\sigma_\pi$ being a random number drawn from a normal distribution with a mean of 1 and a standard deviation of 0.1. Such variation may reflect for example individual sampling variation in the number and effect size of loci that bear deleterious alleles, or random environmental effects on individual survival probability.

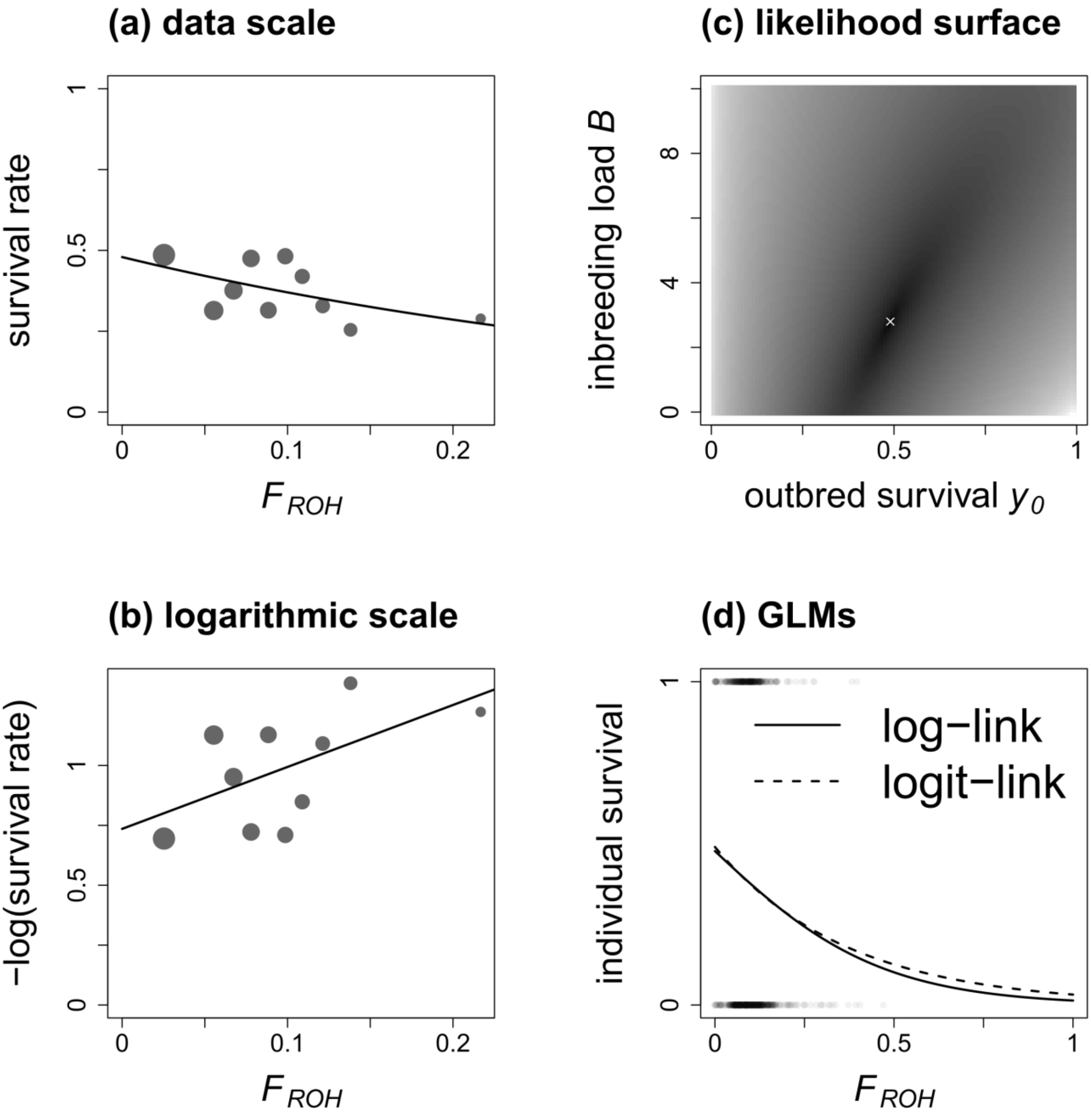


**Figure S4.** Legend on following page.

**Figure S4 (previous page).** Illustration of models for estimation of inbreeding load with data of survival and inbreeding from one simulated representative deme with a sample size of 791 individuals. This deme had a true inbreeding load of $B = 2.01$. The model of Morton et al. (1956) requires that individuals are grouped into classes of inbreeding coefficients, so that mean survival rate can be calculated for each class. **(a)** The relationship between survival rate and inbreeding coefficient is shown on the data scale. **(b)** The regression analysis is performed on a logarithmic scale and revealed $A = 0.74$ and $B = 2.59$. The size of the dots in (a) and (b) is proportional to the weight assigned to each data point (see text in Supporting Information 1 for methodological details). **(c)** Kalinowski and Hedrick (1998) proposed to estimate $y_F = y_0 e^{-BF}$ with $y_0 = e^{-A}$ directly with maximum-likelihood. Here, the likelihood surface is shown with darker colours corresponding to higher likelihoods. The maximum-likelihood is marked with a white cross, and yielded $y_0 = 0.49$, or equivalently $A = 0.71$, and $B = 2.80$. **(d)** Individual survival as a binary variable is used in the analysis with generalized linear models, with logarithmic link function and Poisson-distributed errors (solid line) or with logit link function and binomial error distribution (dashed line). A GLM with Poisson errors and logarithmic link yielded $A = 0.61$ and $B = 2.53$. A GLM with binomial errors and a logit link yielded $A = 0.74$. Estimates of $B$ from such a GLM depended on the arbitrary choice of the two classes of inbreeding coefficients for which survival rate was predicted. For example, when using $F_0 = 0$ and $F_i = 0.25$, $B = 2.70$. For $F_0 = 0$ and $F_i = 0.5$, $B = 3.10$; for $F_0 = 0$ and $F_i = 1$, $B = 3.54$; and for $F_0 = 0.5$ and $F_i = 1$, $B = 3.99$.

# ***Results***

We compared mean estimates of inbreeding load $B$ across all simulation replicates for $F_{ROH}$, using the four models illustrated in Figure S4 and summarised in Table 2. Using equation 1 to extract the actually present inbreeding load yielded a mean of $B = 1.83$ across all 280 replicates. The model of Morton et al. (1956) estimated $B = 1.68$, both with and without applying the small sample size correction proposed by Templeton and Read (1983, 1984). The rather small inbreeding load resulted in all inbreeding classes containing a sufficient number of survivors so that the small sample size correction affected inbreeding load by less than $10^{-5}$ lethal equivalents. The maximum-likelihood model proposed by Kalinowski and Hedrick (1998) yielded $B = 1.85$. A GLM with Poisson errors and logarithmic link yielded $B = 1.86$. Estimates of $B$ from a GLM with binomial errors and logit link depended on the arbitrary choice of the two classes of inbreeding coefficients for which survival rate is predicted. For example, when using $F_0 = 0$ and $F_i = 0.25$, $B = 1.87$. For $F_0 = 0$ and $F_i = 0.5$, $B = 2.13$; for $F_0 = 0$ and $F_i = 1$, $B = 2.43$; and for $F_0 = 0.5$ and $F_i = 1$, $B = 2.74$. These differences of $B$ from the same model are large, not desirable, and cannot be ameliorated by resorting to a proposed arbitrary “convention” (Grueber et al. 2011, page 710) of using $F_0 = 0$ and $F_i = 0.25$. The shape of the logistic regression line (Figure S4d) prevents comparability of such estimates between studies.

# Supporting Information 2: Metrics of *F*, subsets of loci or pedigree data, and fewer individuals

## *Methods*

We illustrate the different metrics of *F* by plotting their values observed in one deme (Figure S5), and we report some summary statistics for them in Table 3. To investigate the effects of the amount of genetic data used to calculate *F* on estimates of inbreeding load, we also calculated inbreeding coefficients based on several restricted datasets. $F_{ped}$ was also calculated with only three to seven known ancestral generations. $F_H$ and $F_{alt}$ were also calculated for randomly selected sets of 25,000, 10,000, 5,000, and 1,000 polymorphic loci. $F_{ROH}$ was also calculated using only runs of homozygosity of at least 0.5, 2, or 5 Mbp in length, corresponding to coalescence events occurring on average 49.0, 12.3, or 4.9 generations ago, respectively (see main manuscript for calculations). To more closely investigate $F_H$ and its dependence on allele frequencies, we calculated $F_H$ based on three more sets of loci: (i) all polymorphic deleterious loci; (ii) using a subset of neutral loci that most closely matched the allele frequencies of the deleterious loci; and (iii) using the subset of neutral loci with relatively even allele frequencies with a minor allele frequency of at least 0.2. To investigate the effects of sample size, we also calculated inbreeding load using the full genetic dataset but only subsets of 500 or 250 individuals that we randomly selected from the ca. 800 individuals of the four focal generations that formed the main dataset for each deme.

## *Results*

Figure S5 shows how the different metrics of *F* differ from each other. We observed that *F* of immigrants (red dots in Figure S5; immigrants were excluded from all other analyses) were similar to *F* of individuals born in the focal deme for $F_{ped}$, $F_{ROH}$ and $F_H$, but they were mostly much higher for $F_{alt}$. Higher $F_{alt}$ of immigrants is a consequence of the high weight given to homozygotes of a rare allele. Because different demes in a metapopulation typically have different alleles at low frequencies due to genetic drift (e.g. Crow and Kimura 1970, chapter 2.9), immigrants get very high values of $F_{alt}$ even when they are not more inbred than individuals born in the focal population. A similar effect appears to also produce positive values of $F_{alt}$ of several individuals with one immigrant parent (blue dots in Figure S5). Such individuals are normally the least inbred individuals and their *F* should thus show values close to the low end of the distribution, as is the case for $F_{ped}$, $F_{ROH}$ and $F_H$.

As shown in Figure S6, using runs of homozygosity longer than 0.5 yielded unbiased estimates of inbreeding load, whereas runs of homozygosity longer than 5 Mbp yielded an overestimate. Use of $F_H$ underestimated inbreeding load more strongly with smaller subsets of neutral loci. Use of $F_{alt}$ overestimated inbreeding load, but decreasingly so with smaller subsets of neutral loci. We also investigated how well $F_H$ performed when calculated using only deleterious loci (situation (i) in Methods section above). $F_{H(del)}$ strongly underestimated inbreeding load, even though it was calculated across all loci with effects on survival. As discussed and justified in the Supporting Information 4 and Figure S11, this is likely due to the fact that deleterious alleles occur at rather low frequencies which have low power to predict genomic rates of homozygosity and identity by descent. This is supported by the observation that neutral loci with allele frequencies matched to the deleterious loci (situation (ii) in Methods section above) were basically useless for estimation of inbreeding load, yielding an estimate close to 0. Loci with relatively even allele frequencies (situation (iii) in Methods section above) on the other hand led to overestimated inbreeding load (Figure S6).

We further investigated what effect sample size had by calculating inbreeding load with subsets of 500 or 250 randomly selected individuals (Figure S6) out of the ca. 800 individuals forming the datasets of each deme. Using smaller sample sizes did not affect mean estimates of inbreeding load across all replicates, but increased the spread of estimates for all inbreeding coefficients. This suggests that even when using much larger datasets and sufficient genetic data, $F_H$ and $F_{alt}$ would still yield biased estimates of inbreeding load, whereas $F_{ROH}$ would yield unbiased estimates of higher precision.

Figures S7 to S10 impose different cutoffs for loci included in estimation of $F$. Using only loci with a minor allele frequency of more than 5% had some quantitative effects on our results, but did not qualitatively affect any conclusions. The values of $F_{alt}$ for immigrants and their descendants were still higher than expected (see above for expectations), but less extremely so than when not imposing a minor allele frequency cutoff (Figure S7). Biases in estimates of inbreeding load remained similar (Figure S8). Additionally pruning loci in high linkage disequilibrium (using the same settings as described in the main text for $F_{ROH}$), did not affect the results (Figures S9 and S10).

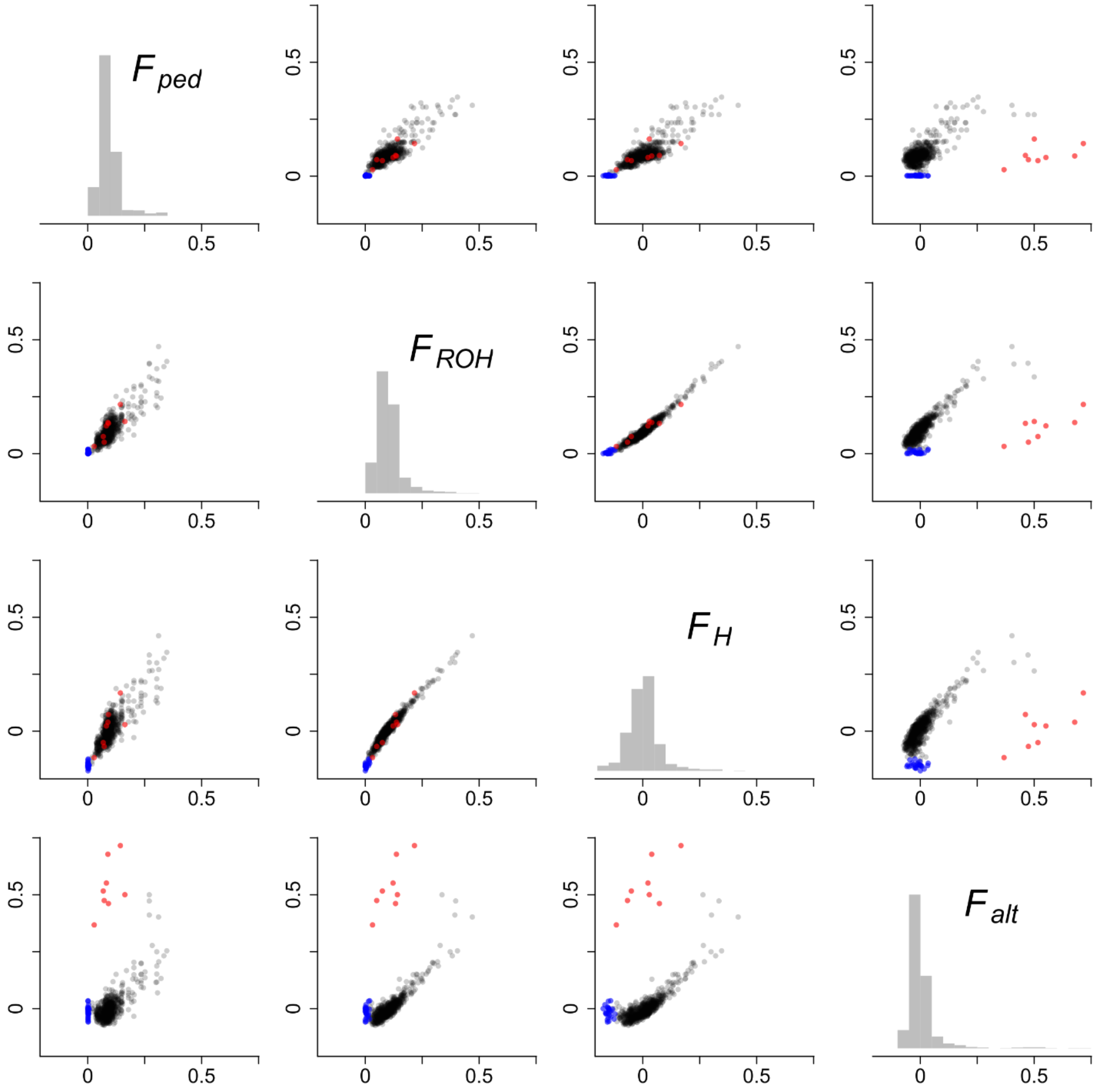


**Figure S5.** Distributions and pairwise relationships of different metrics of *F* using illustrative data from one simulated deme. The axes correspond to the metric of *F* printed in the respective row or column. Red dots indicate immigrants. Immigrants were excluded from all other analyses in this manuscript. Blue dots represent individuals with one immigrant parent. Grey dots show all other individuals born in the focal deme and with parents that were also both born in the focal deme. Inbreeding coefficients shown here were calculated with the largest available simulated datasets and $F_{ROH}$ used runs of homozygosity larger than 1 Mbp.

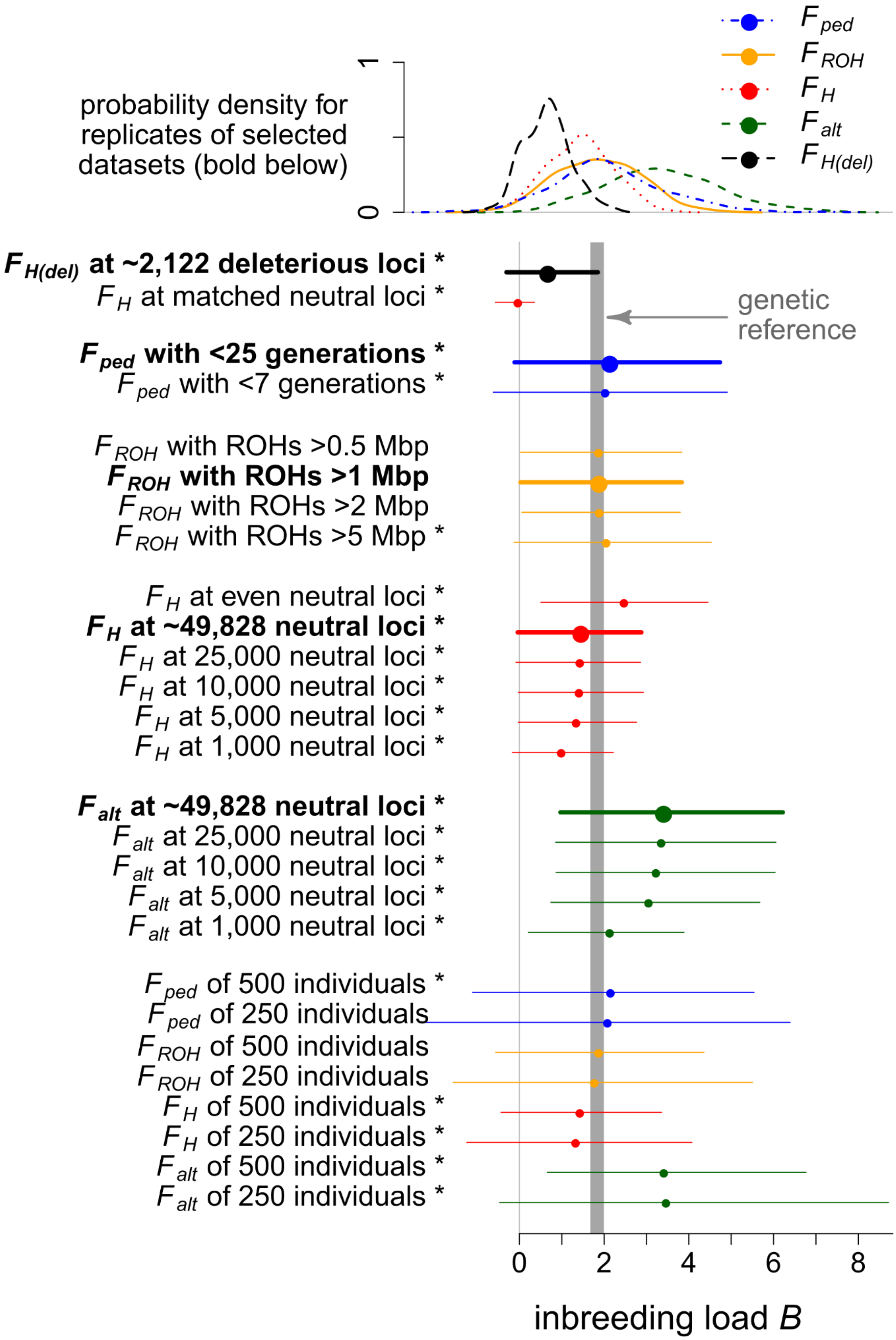


**Figure S6.** Legend on following page.

**Figure S6 (previous page).** Inbreeding load estimated in a Poisson GLM with logarithmic link function and various methods for estimating inbreeding coefficients (see main text for details). Curves on top of the panel show probability densities for the inbreeding coefficients based on all recorded genetic markers or pedigree generations. Horizontal lines in the lower part of the panel show the 2.5% to 97.5% quantiles, and dots indicate mean estimates across all 280 replicates. Asterisks (*) indicate that the mean estimate was different from the true value of inbreeding load with a p-value of less than 5%. $F_{H(del)}$ represents $F_H$ calculated across all deleterious loci (black). The thin red line below the black line represents $F_H$ calculated from those neutral loci that matched the allele frequencies of the deleterious loci most closely. $F_{ped}$ (blue) was based on up to 25 (thick line) or up to 7 (thin line) ancestral generations. $F_{ROH}$ (orange) was based on runs of homozygosity of at least 1 Mbp (thick line) or at least 0.5, 2, or 5 Mbp (thin lines). $F_H$ (red) was calculated using all polymorphic neutral loci (thick line), or a subset of 25,000; 10,000; 5,000; or 1,000 randomly selected loci (thin lines); or based on the subset of neutral loci with minor allele frequencies larger than 0.2 (“at even loci”). $F_{alt}$ (green) was calculated using all polymorphic neutral loci (thick line), or a subset of 25,000; 10,000; 5,000; or 1,000 randomly selected loci (thin lines). The area shaded in grey shows the genetic reference of actual inbreeding load as calculated from the observed allele frequencies and selection coefficients at deleterious loci using equation 1. The lowest eight lines are calculated with random subsets of individuals and using all available genetic data.

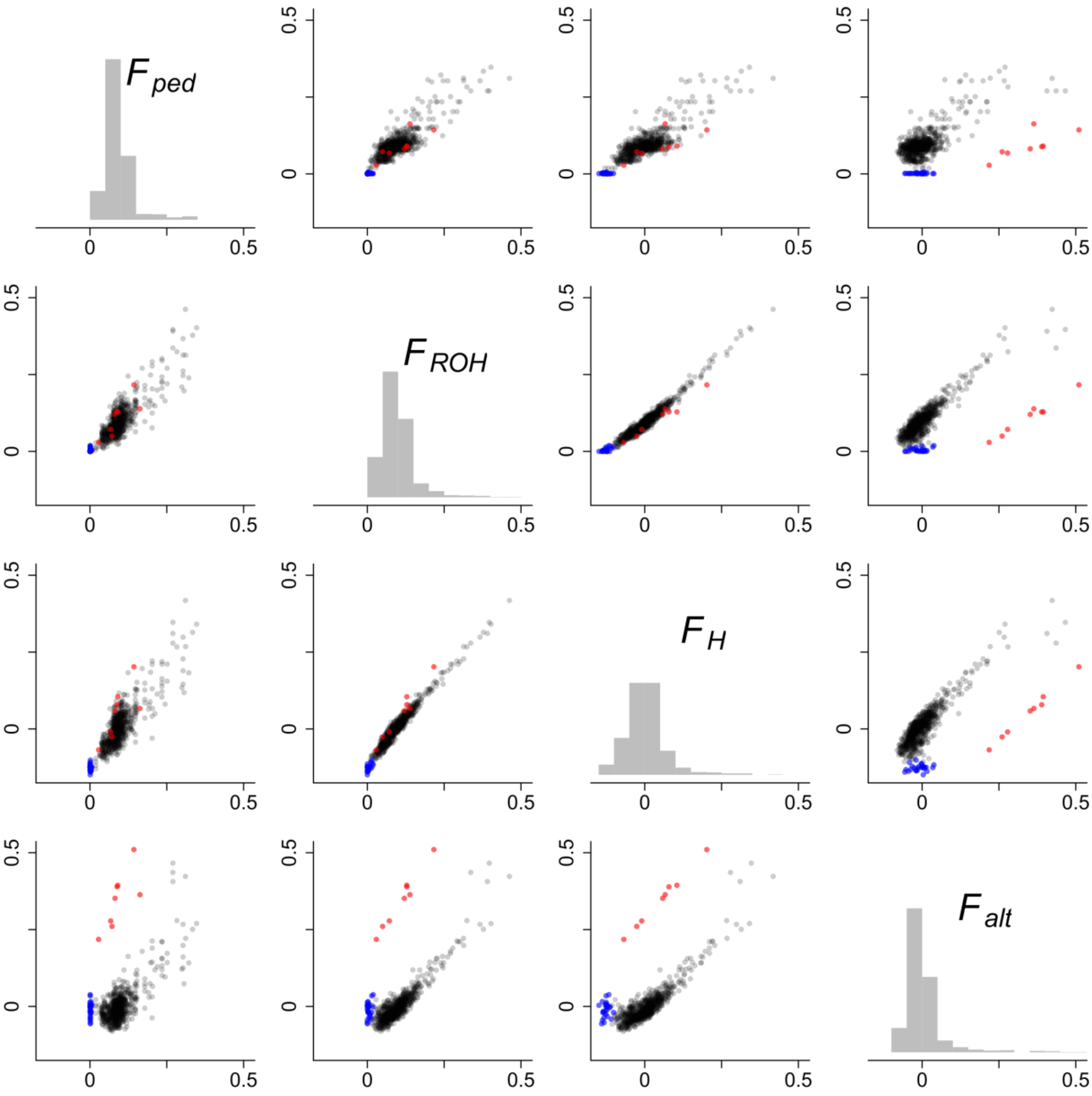


**Figure S7.** Distributions and pairwise relationships of different metrics of *F* using illustrative data from one simulated deme. Here, only those neutral loci with a minor allele frequency higher than 5% were included; this is the difference to the data shown in Figure S5 (see the legend there for more details).

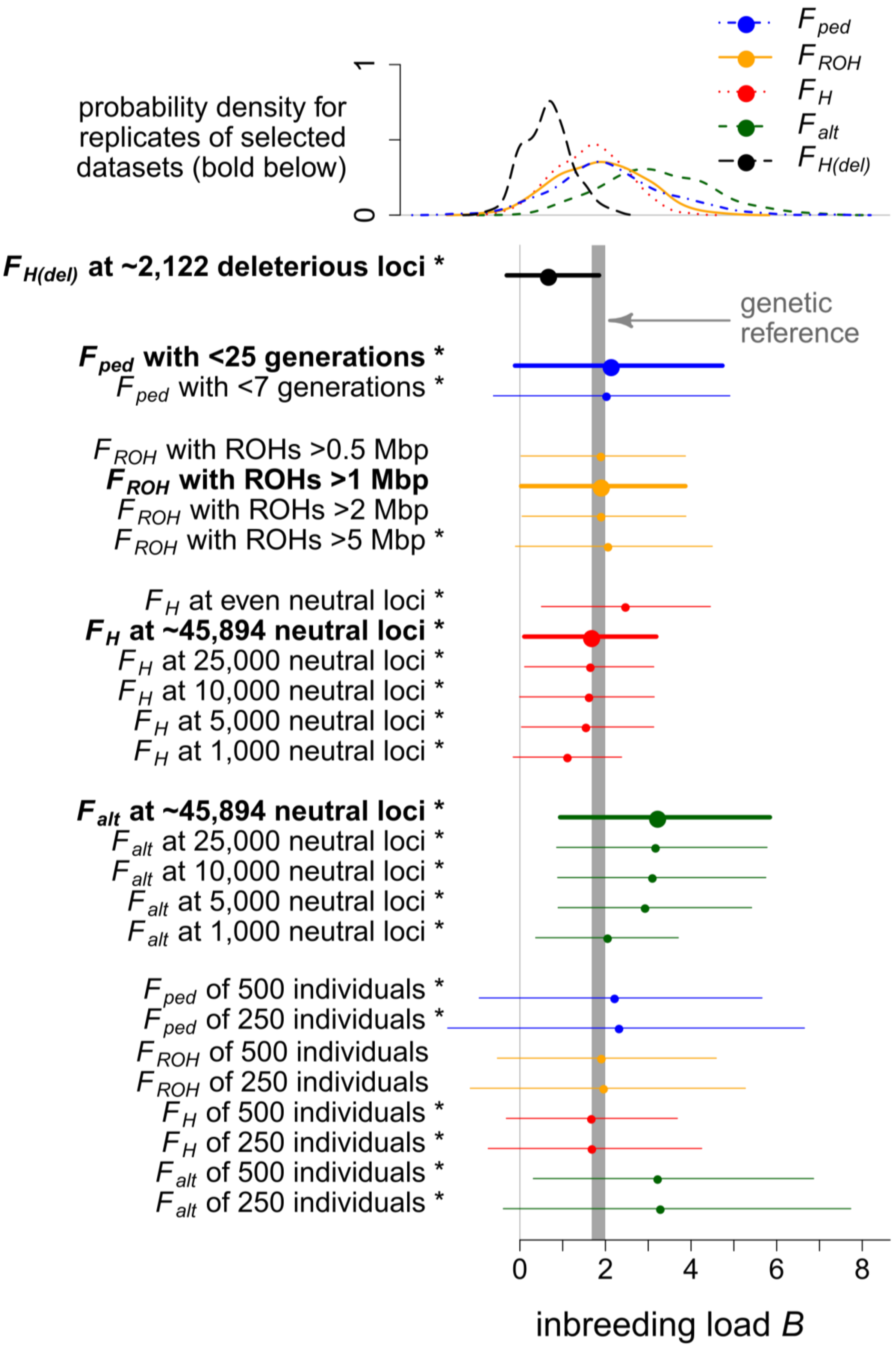


**Figure S8.** Same plot as in Figure S6 (see legend there for explanations), except that only those neutral loci with a minor allele frequency higher than 5% were included.

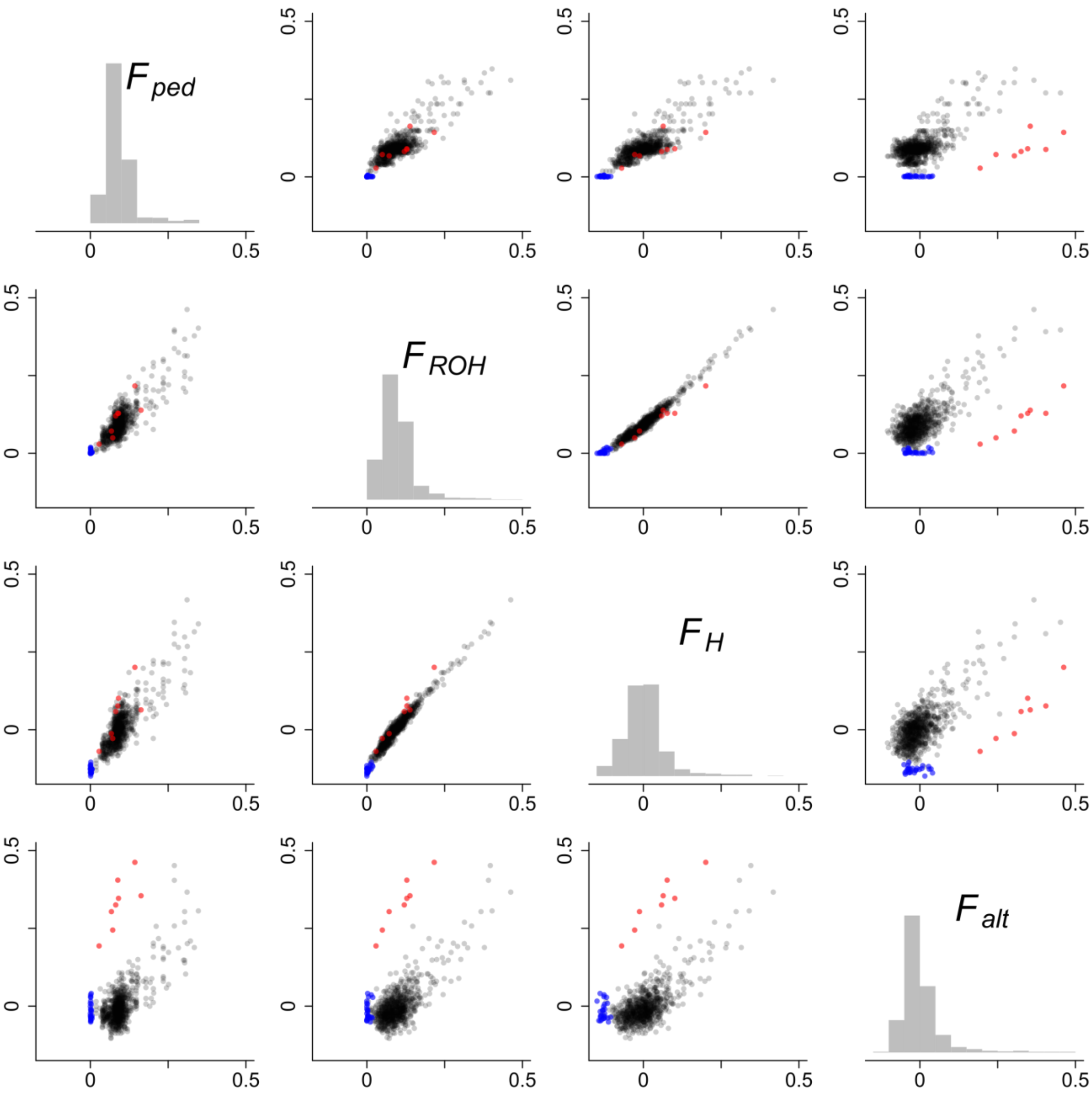


**Figure S9.** Distributions and pairwise relationships of different metrics of *F* using illustrative data from one simulated deme. Here, only those neutral loci with a minor allele frequency higher than 5% were included, and loci in strong linkage disequilibrium were pruned; these are the differences to the data shown in Figure S5 (see the legend there for more details).

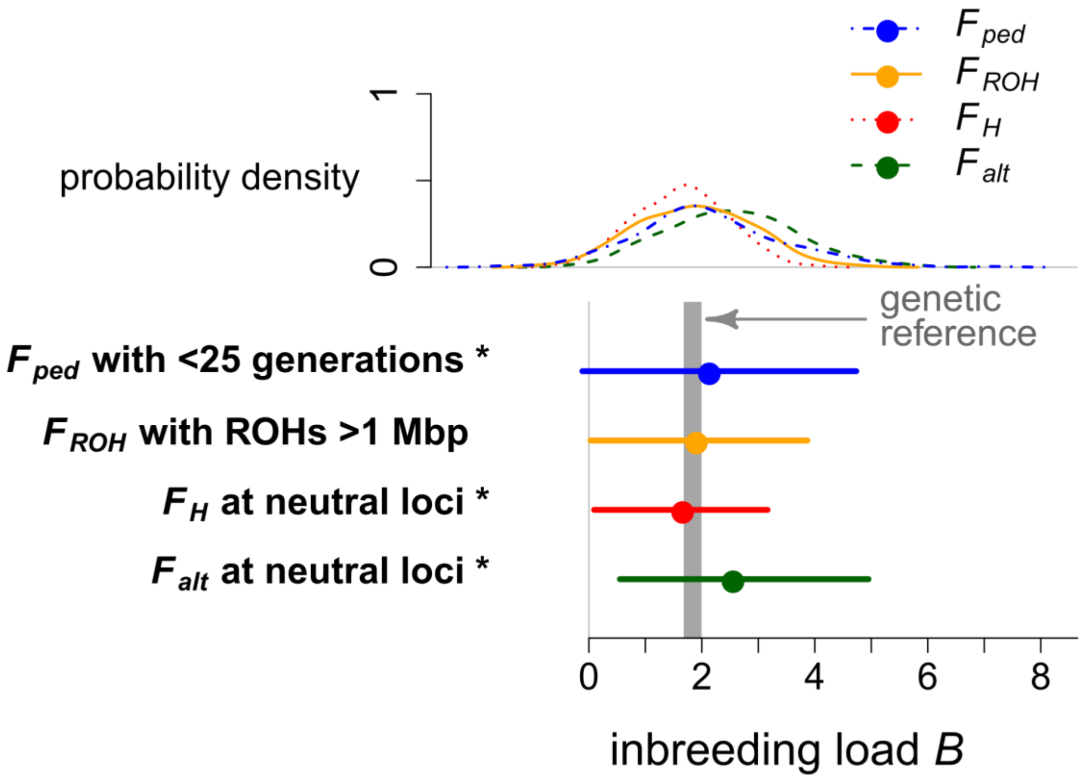


**Figure S10.** Same plot as in Figure 2 of the main manuscript (see legend there for explanations), except that only those neutral loci with a minor allele frequency higher than 5% were included, and linkage disequilibrium pruning was conducted for all metrics of *F*.

# Supporting Information 3: Heterozygosity or probability of identity-by-descent for prediction of survival

For the genomic simulations described in the main manuscript, we explicitly simulated genotypes at loci with deleterious mutations. The genotypes at these loci were the cause of fitness effects. In this simulation setup, realized identity-by-descent is not known for individual loci, but it is also not necessary to be known because fitness consequences are directly caused by the simulated genotypes. In reality, the loci causing fitness consequences are typically not or not entirely known, and inbreeding depression is typically studied by using neutral loci or loci with unknown fitness effects to estimate inbreeding coefficients, as we have done here. Nonetheless, the implicit assumption is that estimates of genome-wide identity-by-descent correlate with heterozygosity at loci with fitness effects (i.e. deleterious loci). Thereby, one might expect that knowing the actual levels of heterozygosity at deleterious loci would explain more variation in fitness than genome-wide levels of heterozygosity. We show here in some simplistic simulations that although this expectation holds for many situations, it does not hold when the deleterious allele is very rare.

In our simulations, $F_{ped}$, $F_H$, and $F_{ROH}$ all correlated similarly well with individual survival with mean correlation coefficients of -0.07 to -0.06. Correlations of survival with $F_H$ at deleterious loci only (i.e., $F_{H(del)}$) was much weaker with a mean correlation coefficient of -0.04. This finding was initially surprising because the deleterious loci are directly causing variation in survival and information about heterozygosity at these loci was thus expected to explain most variation in survival. Note that $F_{H(del)}$ is perfectly correlated with heterozygosity at deleterious loci. Deleterious loci are under selection and as a consequence, their mean allele frequency of 7.8% across all 280 replicates and mean heterozygosity of 0.12 are rather low. In such cases, homozygosity (i.e. identity-by-state, IBS) is not very informative about identity-by-descent (IBD) even at these loci themselves. This information content can be quantified as IBD-IBS-discrepancy (Knief et al. 2017). Higher values of IBD-IBS discrepancy correspond to marker IBS being a bad predictor of marker IBD. Highly variable and more heterozygous markers (i.e. more alleles of similar frequency) lead to lower values of IBD-IBS discrepancy because marker variability increases informativeness about IBD (Knief et al. 2017; Nietlisbach et al. 2017). Mean IBD-IBS discrepancy was 0.86 at deleterious loci, and 0.60 at neutral loci.

To investigate if high IBD-IBS-discrepancy might be the reason for the low correlation of $F_{H(del)}$ with survival, we conducted explorative simulations across the whole range of deleterious allele frequencies. We simulated genotypes at a single recessive lethal locus for individuals with a range of inbreeding coefficients. These genotypes were then used to calculate survival probabilities and then to simulate actual survival data. We then investigated how much variation in survival was explained by heterozygosity at the single deleterious locus (red dots in Figure S11), or by the underlying inbreeding coefficients (blue dots in Figure S11), i.e. by the genome-wide probability of identity-by-descent used to simulate the genotypes at the deleterious locus. We found that when allele frequencies were very low, the underlying genome-wide probabilities of identity-by-descent explained slightly more variance in survival than heterozygosity at the causal locus. Thus, with high IBD-IBS-discrepancy and low deleterious allele frequency, good knowledge of identity-by-descent appears to be more useful to predict survival than knowledge of heterozygosity at the causal deleterious locus. IBD-IBS discrepancy is also high when the deleterious recessive allele is very common, but then heterozygosity is highly correlated with survival, because heterozygous genotypes differ in their fitness effects from the genotypes that are homozygous for the deleterious recessive allele (which is the most common genotype when deleterious allele frequency is high). Note that the discussion in this section of the Supporting Information focused on correlation coefficients or their squared value (i.e. the variance explained), because they are not affected by different ranges and variances of the various inbreeding coefficients.

These simulations (Figure S11) suggest that it is likely that high IBD-IBS-discrepancy at the deleterious loci and low frequency of deleterious recessive alleles in our simulations are the reason for why $F_{H(del)}$ is only weakly correlated with individual survival. In support of this, very little variance in survival is explained by heterozygosity at the subset of neutral loci that match allele frequencies of deleterious loci most closely, and $F_H$ across these loci provided practically no information about inbreeding load (i.e. a mean estimate close to 0 as shown for "$F_H$ at matched neutral loci" in Figure S6). This is not a consequence of a smaller number of loci, as even smaller random subsets of neutral loci provided better estimates of inbreeding load. These results suggest that it may be more useful for quantifying the effects of inbreeding if genome-wide realized identity-by-descent is quantified (e.g. through measuring $F_{ROH}$) than if heterozygosity at all deleterious loci was known.

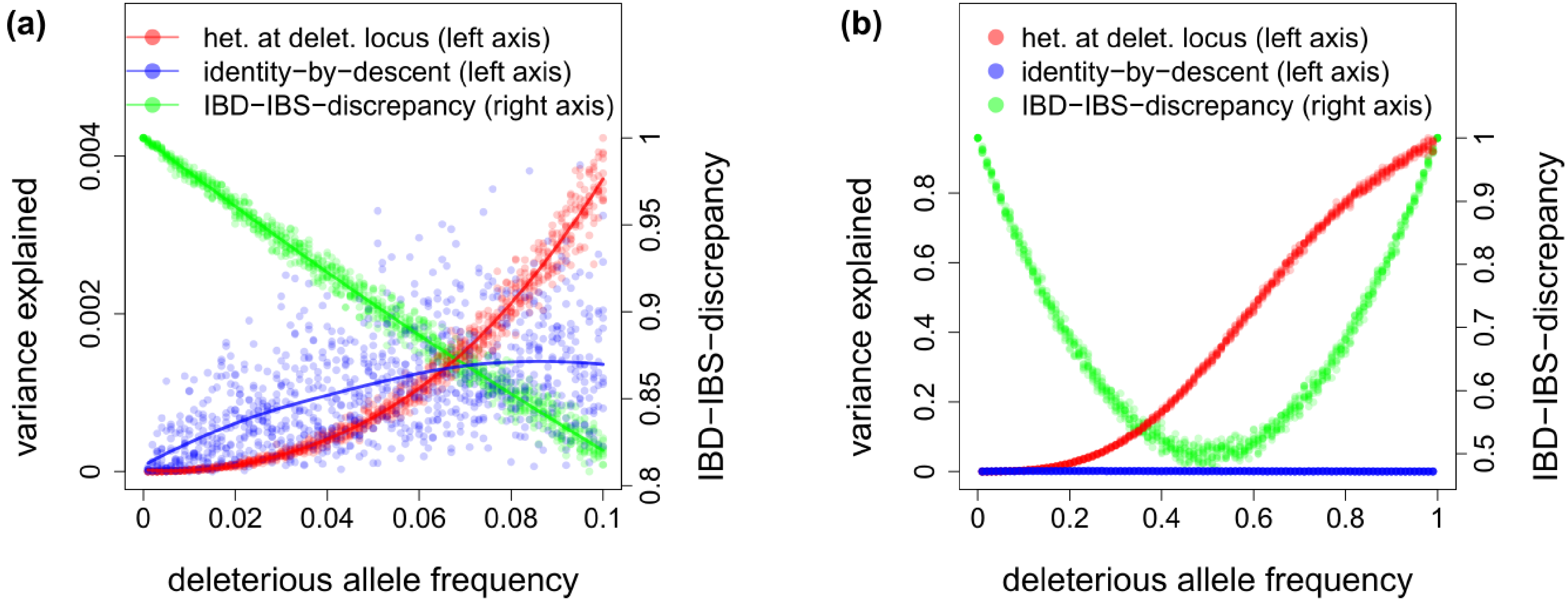


**Figure S11.** Simulations of survival determined by a single recessive lethal locus, whose genotype was determined by a range of inbreeding coefficients that represent measures of genome-wide identity-by-descent. See text of the Supporting Information 3 for more information. Panel **(a)** shows a small section of panel **(b)** for rare deleterious allele frequencies.

# Supporting Information 4: Additional Figures

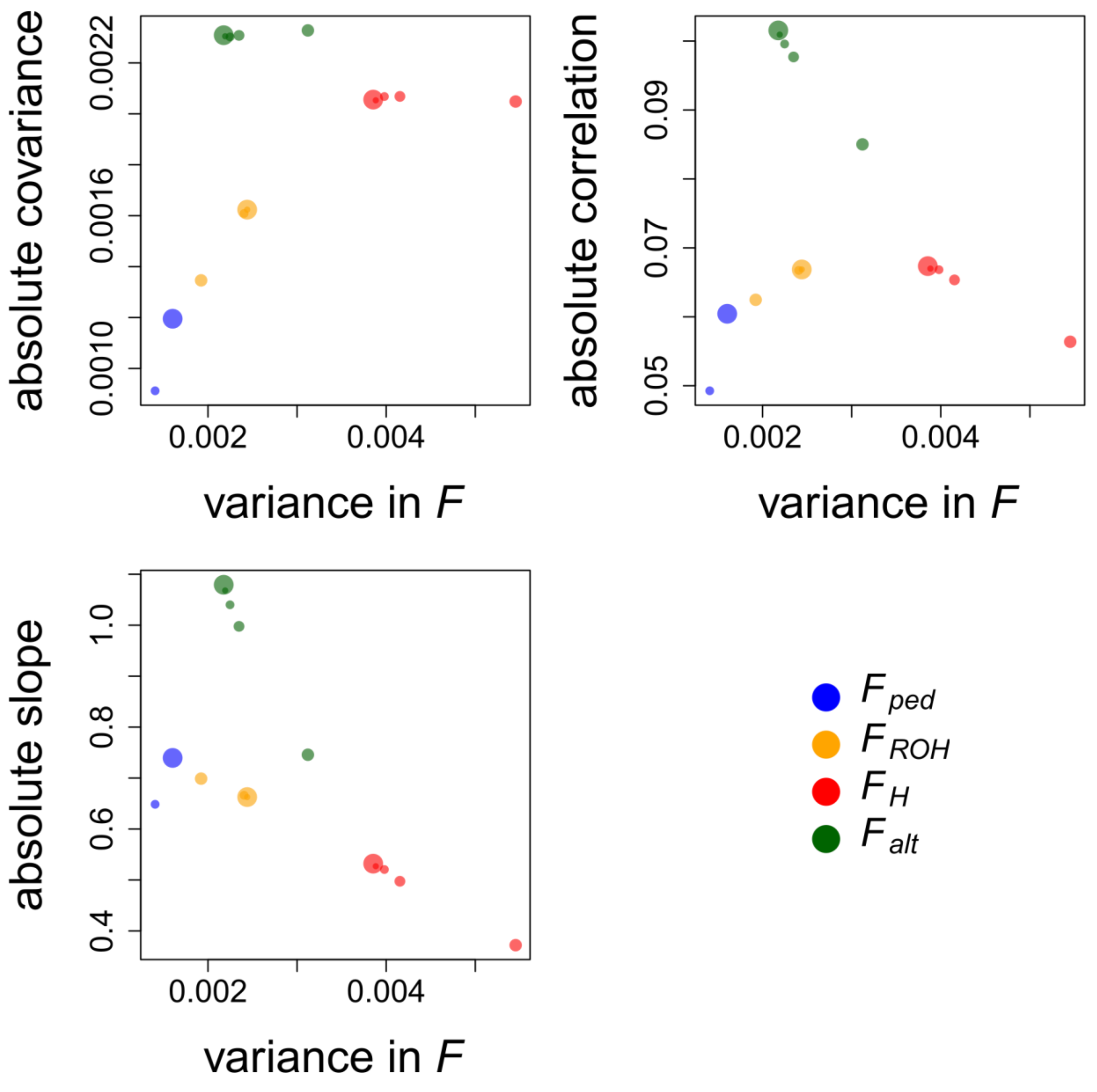


**Figure S12.** Comparison of statistical properties of inbreeding coefficients and their relationships with survival. Absolute covariance, correlation, and slope of observed survival on inbreeding coefficient, and variance in inbreeding coefficient were calculated for each replicate. Means across all replicates are plotted here, with large dots representing the largest available dataset (bold labels in Figure S6 in Supporting Information 2). Smaller dots represent subsets of loci or different minimum length requirements for $F_{ROH}$. Note that here slopes of a regression of survival on inbreeding coefficients are plotted (i.e. no logarithmic relationship), as this allows for direct comparison with covariances and variances, because the regression slope equals the covariance divided by the variance in the independent variable.

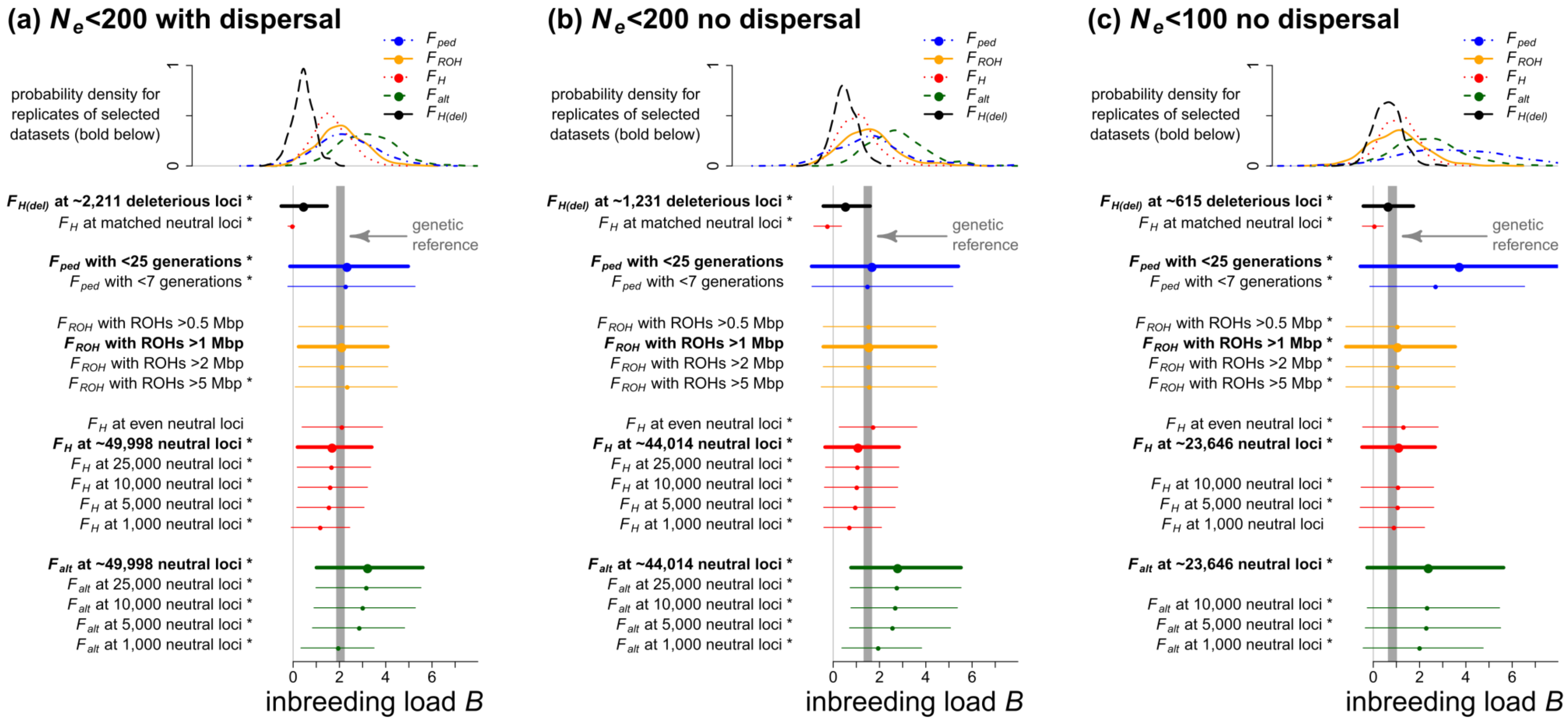


**Figure S13.** Legend on following page.

**Figure S13 (previous page).** Estimates of inbreeding load from simulations run for 100 generations with increasing degree of inbreeding. Plots are analogous to Figure S6 in Supporting Information 2 (see there for further information). **(a)** Simulations were identical to those presented in the main manuscript except that only 100 generations were simulated. **(b)** No dispersal occurred in an otherwise identical simulation as in (a), i.e. with a population size per deme of up to 200 individuals. **(c)** No dispersal occurred in an otherwise identical simulation as in (a), but population size per deme was limited to less than 100 individuals, and settings for calculating runs of homozygosity were modified to allow for lower density of polymorphic neutral loci (at least 1 polymorphic locus every 250 kbp instead of every 100 kbp) and larger stretches without polymorphic loci (up to 4 Mbp instead of 2 Mbp). This was necessary because much fewer loci remained polymorphic in the smaller and isolated demes (see panels). In addition, sample size was decreased to a mean of only 414 individuals per replicate, thus limiting the reliability of the results plotted in (c). Mean sample sizes were 788 for (a), and 800 for (b). In addition, many demes in (c) were extinct or on the path towards extinction. To account for this, we ran simulations for 20 replicate sets of demes, and only used demes with at least 15,000 polymorphic neutral loci and at least 300 individuals in the dataset. This resulted in 248 replicate demes used in (c).

# References in Supporting Information

Armstrong, D. P., and P. Cassey. 2007. Estimating the effect of inbreeding on survival. Animal Conservation 10:487–492.

Crow, J. F., and M. Kimura. 1970. An introduction to population genetics theory. The Blackburn Press, Caldwell, United States of America.

Efron, B., and R. J. Tibshirani. 1993. An introduction to the bootstrap. Chapman & Hall, New York, United States of America.

Glémin, S., L. Vimond, J. Ronfort, T. Bataillon, and A. Mignot. 2006. Marker-based investigation of inbreeding depression in the endangered species *Brassica insularis*. Heredity 97:304–311.

Grueber, C. E., S. Nakagawa, R. J. Laws, and I. G. Jamieson. 2011. Multimodel inference in ecology and evolution: challenges and solutions. Journal of Evolutionary Biology 24:699–711.

Kalinowski, S. T., and P. W. Hedrick. 1998. An improved method for estimating inbreeding depression in pedigrees. Zoo Biology 17:481–497.

Knief, U., B. Kempenaers, and W. Forstmeier. 2017. Meiotic recombination shapes precision of pedigree- and marker-based estimates of inbreeding. Heredity 118:239–248.

Morton, N. E., J. F. Crow, and H. J. Muller. 1956. An estimate of the mutational damage in man from data on consanguineous marriages. Proceedings of the National Academy of Sciences of the United States of America 42:855–863.

Nietlisbach, P., L. F. Keller, G. Camenisch, P. Arcese, J. M. Reid, and E. Postma. 2017. Pedigree-based inbreeding coefficient explains more variation in fitness than heterozygosity at 160 microsatellites in a wild bird population. Proceedings of the Royal Society B 284:20162763.

R Core Team. 2015. R: A language and environment for statistical computing. R Foundation for Statistical Computing, Vienna, Austria.

Templeton, A. R., and B. Read. 1983. The elimination of inbreeding depression in a captive herd of Speke's gazelle. Pages 241–261 *in* C. Schonewald, S. M. Chambers, B. MacBryde, and L. Thomas, eds. Genetics & Conservation: a reference for managing wild animal & plant populations. The Blackburn Press, Caldwell, United States of America.

Templeton, A. R., and B. Read. 1984. Factors eliminating inbreeding depression in a captive herd of Speke's gazelle (*Gazella spekei*). Zoo Biology 3:177–199.

Zeileis, A. 2004. Econometric computing with HC and HAC covariance matrix estimators. Journal of Statistical Software 11:1–17.

———. 2006. Object-oriented computation of sandwich estimators. Journal of Statistical Software 16:1–16.

Zeileis, A., and T. Hothorn. 2002. Diagnostic checking in regression. The Newsletter of the R Project 2:7–10.

Zou, G. 2004. A modified Poisson regression approach to prospective studies with binary data. American Journal of Epidemiology 159:702–706.
